# If you move, I move: The social influence effect on residential mobility

**DOI:** 10.1371/journal.pone.0270783

**Published:** 2022-07-06

**Authors:** Àlex G. de la Prada, Eduardo Tapia

**Affiliations:** Institute for Analytical Sociology, Department of Management and Engineering, Linköping University, Norrköping, Sweden; Bruno Kessler Foundation, ITALY

## Abstract

There are many theories that account for why households move between residential areas. In this paper, we advance on this by formulating a new mechanism whereby a household’s probability of leaving a neighborhood is informed by the number of other households who have previously left that neighborhood. We call this mechanism: the social influence (SI) effect. By applying matching to Swedish register data for Stockholm County (1998–2017), and after adjusting for theoretically relevant confounders from the existing literature, we find that SI has a significant effect on neighborhood out-mobility. Furthermore, we find that the SI effect is moderated by the visibility with which others’ behaviors is observed, measured as the number of previous out-movers, the distance to ego, and its salience in the social environment. Our study also discusses some ways in which SI might be entangled with other mechanisms, and outlines future directions from which studies of residential segregation dynamics might be approached.

## Introduction

Either ethnically or socioeconomically colored, segregation scholars suggest that the spatial separation of groups has negative consequences for the welfare of segregated families, as it fosters an unequal distribution of opportunities across residential areas. For example, households that are overrepresented in economically disadvantaged neighborhoods [1] are at increased risk of ending up with lower earnings and of suffering from industrial hazards [2–6].

Social scientists agree that households’ selective mobility patterns—leaving and moving into different neighborhoods—constitute the main motor of residential segregation. Previous studies have reported these mobility patterns to be determined by distinct factors, including households’ life-course events, housing market characteristics, and households’ neighborhood preferences [7–11]. However, previous research has largely overlooked the possibility that individuals’ mobility decisions are affected by others’ past mobility behavior: the social influence (SI) effect.

Studying how individuals’ behavior is influenced by the past behavior of their peers has been a cross-sectional topic of inquiry in the social science literature. It has allowed researchers to better understand individual choices beyond personal preferences and socioeconomic characteristics. In addition, SI-related studies provide a theoretical framework for the sociological understanding of how some macro events are linked to micro behaviors.

The generalizability of this framework has permitted its application to a wide range of sociological phenomena [12–14]. For instance, Hedström [15] has observed how people previously having joined unions increases the chances of others joining, allowing trade unions to spread spatially. In the context of cultural markets, Salganik, Dodds and Watts [16] show that prior information on the popularity of songs, compared to a blinded situation, increases the unpredictability and inequality of their market share (see also [17–19]. Despite the substantial amount of empirical evidence showing the effect of SI in relation to a wide variety of issues, to our knowledge, no attempt has been made to investigate whether SI plays a role in neighborhood choice.

Building on social theories of influential behavior as a belief-formation process [20] and self-attention theory [21], we hypothesize that, after adjusting for factors that have previously been identified as significant in the literature on neighborhood mobility, SI will play a role in whether households leave or stay in their current neighborhoods. (We are aware that SI can also influence in-mobility. However, studying this would require a different approach to that taken here, and also a different dataset.) More concretely, our theoretical argument states that when a household leaves a neighborhood, it sends a negative signal (leaving the neighborhood) to other neighbors about the neighborhood that is being left. As a result, this negative signal contributes to a negative change in neighbors’ beliefs about the desirability of the current neighborhood, which in turn increases their own likelihood of moving-out.

As in previous studies of SI [16], we also hypothesize that the strength of this effect will be moderated by the ease with which the signal is perceived by the receivers in the following respects: (1) the number of households leaving the neighborhood; (2) the residential density of the area; (3) the physical distance between the signal and the receiver, and (4) the proportion of non-natives living in a neighborhood.

This study examines how the earlier residential mobility behavior of households influences the moving-out behavior of current residents, adjusting for previous explanations found in the literature on neighborhood mobility. Our analysis focuses on register data for Stockholm County. Apart from providing long time-series in relation to immigration processes, the granularity of Swedish registers provides a unique opportunity to study the effect of SI, since it allows us to control for other important factors relating to neighborhood mobility. However, despite the richness of the data, the lack of a significant number of non-native cases for all of our confounder variables has forced us to restrict our focus to studying the native population.

By applying Coarsened Exact Matching (CEM), we exploit variation in natives’ exposure only to out-movers or to an absence of movers in order to assess the effect of SI on out-mobility behavior, and also to evaluate how the visibility of influential signals tempers, or exacerbates, the SI effect. Our results show that SI plays a key role in relation to residential out-mobility. Specifically, we find that households are more likely to leave neighborhoods when other households have previously left, compared to when there has been no such change. In addition, we also find that this effect is stronger when previous out-movers leave lightly populated areas, are physically closer, and greater in number. We found no evidence for an effect of neighborhood ethnic composition.

This study contributes to the literature on residential mobility in two ways. First, the study of social influence allows us to evaluate the effect of one of the most intriguing and relevant topics in social science research in a novel context where it has not previously been tested. Second, we are not only proposing a new causal mechanism that drives neighborhood mobility and, consequently, residential segregation patterns, but also advancing the theory of social influence itself by providing new empirical evidence on its occurrence and intensity.

The structure of this paper is as follows. First, we introduce current theories that account for residential mobility, and this is followed by an introduction on SI theories. We then introduce our analytical design and explain how we apply CEM to Swedish register data. Finally, we present and discuss our results. We conclude this paper with a discussion.

### Neighborhood mobility

Residential segregation is an important issue in modern societies. Scholars have repeatedly documented its detrimental effects in a wide variety of contexts (e.g., [1–4, 6, 22–24]).

Previous studies have noted that patterns of segregation are driven by the ways in which households move around cities. Depending on the scientific field, several explanations have been provided to account for why and how households move between neighborhoods. For instance, demographic explanations focus on households’ dwelling requirements and the shifting role of these requirements in the life-trajectories of families. These explanations argue that life-course events play an important role for mobility because these events shift and unbalance households’ space-related housing needs, motivating the search for new dwellings [7]. Thus, changes in family composition, such as getting married or having children, have been found to be important predictors of mobility [25, 26]. Similarly, age, socioeconomic status, and tenure position have also been found to have important effects on neighborhood mobility. Hence, younger, renters, and affluent households are more likely to move out than their counterparts [8, 10]. The length of staying in neighborhoods also matters in residential mobility. For example, Fischer and Malmberg [27] found that individuals living for longer periods in the same local market areas are more likely to remain and not move than those with shorter staying periods given that they are deeply rooted in them through kinship and friendship ties.

On the other hand, sociological explanations instead direct their focus at the interaction between neighborhoods’ environments and households’ socioeconomic-ethnic attributes as a trigger of residential mobility. A significant part of this literature points to the ethnic composition and socioeconomic status of neighborhoods as the main drivers of neighborhood mobility. These explanations state that households hold ideal preferences, in terms of neighborhoods’ ethnic composition and socioeconomic status, with regard to where they want to reside. Mobility is thus a response to the desire to fulfill these preferences [5, 9, 11, 22, 28, 29].

Within this preference-based explanatory approach, natives typically prefer living in native-dominated neighborhoods whereas non-natives search for more racially mixed areas [30]. As a consequence, residential segregation increases as natives leave and avoid neighborhoods that experience an increase in the share of non-natives (e.g., [31–41]).

In addition to the processes outlined above, scholars have also documented the effect of neighborhoods’ socioeconomic status on households’ mobility. For example, it has been found that improvements in the socioeconomic status of a neighborhood foster the out-migration of low-affluence families [42] and, by contrast, a decline would promote the flight of more affluent families [9, 31]. At the same time, a decline in the quality of buildings or schools has also been documented to produce more high-status households to move-out [5]. Either way, it is high-status households who have greater opportunities to react to changes in their social environments.

While all previous explanations offer different micro-mechanisms to account for moving-out behavior, it remains unclear whether the prior out-mobility (There are other explanations related to inter-neighborhood mobility which have also noted out in previous studies, but which nevertheless are not directly related to the scope of this study. For example, scholars have also shown the presence of discriminatory practices in the housing market (e.g., [43, 44]), which bias the in-flow of non-native families into certain neighborhoods.) of residents can increase the likelihood of out-mobility among former neighbors after adjusting for all of the existing mobility explanations described above. We call this the SI effect. The aim of this study is thus to propose the SI effect as an alternative and complementary explanation for residential mobility.

### The social influence effect

The long-standing relevance of SI in the sociological literature has been due to the way it complements existing models of individual decision-making in a wide range of contexts and, most importantly, as a result of its suitability in disentangling how the effects of such decisions lead to macro-social patterns of interest. For example, Granovetter’s threshold model [12] states that the propensity of individuals to participate in a riot is a function of the number of persons already involved. The implication of this premise is that macro results are highly dependent on how micro actions unfold over time as a consequence of previous actions, bringing about different aggregate results as a function of the sequence in which things happen [45, 46].

In its most general conceptualization, SI refers to the effect of previous individual actions on the future behavior of other individuals who are aware of those actions. Essentially, we claim to observe SI whenever an individual modifies her expected behavior after observing the behavior of others; for example, when the likelihood of X doing A instead of B increases as a result of X having perceived others doing A. This example reveals three elemental properties of SI. (1) At the individual level, people usually follow certain motivations in order to adjust their behavior to the behavior of others. (2) In any SI pattern, there is always a specific action that is first perceived and then imitated. (3) SI takes place as long as others’ behavior is perceived.

Empirical patterns of the type described in the above example can be driven by several mechanisms at the individual level. Distinct psychological mechanisms have been argued to account for why individuals follow others’ behaviors, such as conformity [47]; compliance [13]; social learning [48]; rational imitation [20]; or legitimacy [49], to name a few examples. In this paper, we conceive of SI as a belief-formation process [20]. Concretely, we argue that an individual who leaves a neighborhood sends a negative signal to other neighbors about the desirability of the neighborhood, which in turn undermines the current favorable belief of neighbors regarding the area in question, thus increasing their likelihood of moving-out.

Previous studies have found similar patterns in other domains. For example, in the context of labor market mobility, Felps et al. [50] find that the probability of a worker quitting her job is higher when other co-workers have previously quit. Likewise, we hypothesize that the probability of a household leaving a neighborhood is greater when (an)other household(s) has/have previously left, as compared to a situation in which nobody has left.

While the previous hypothesis is related to the existence of SI in relation to neighborhood mobility, the following section elaborates on how the strength of SI can vary depending on how likely it is that the receivers receive the signal. More concretely, in this study we identify four factors that moderate the ease with which these signals are received: (1) the number of sources; (2) the residential density of the area; (3) the physical distance to the sources; and (4) the ethnic composition of the area.

The first factor fuels the following hypothesis: the larger the number of previous movers, the greater the probability that others will leave the neighborhood. The rationality of this hypothesis is not only based on the fact that a greater number of movers are more visible than a smaller number, it also captures the idea that a greater number of movers may exert a greater reinforcing impact on the negative signal, as has been shown in several studies on the adoption of innovations (e.g., [12, 16, 17, 19, 51–54]).

The second factor, residential density, also builds on the visibility of out-movers. Thus, we hypothesize that in poorly populated areas the salience of out-movers’ behavior is greater compared to areas of high population density.

The third factor, on the other hand, holds that the greater the distance between the origin of the signal (households leaving the area) and the receiver, the lower the probability that the receivers will leave the area. This factor captures the degree of interaction among individuals distributed in space, and how they influence each other (e.g., [55, 56]). In concrete terms, individuals exert more influence on others the closer they are to them in space [15, 57, 58].

Finally, Self-Attention Theory [21] serves as the basis for the final factor affecting the strength of SI. This theory postulates that in-group attention varies as a function of group composition. More concretely, the lower the proportion of in-group members in the group, the higher the salience of in-group members, and, consequently, the greater the level of attention paid to them [59]. Transferring this to the neighborhood mobility context: natives moving-out from a native neighborhood are less salient to other natives than natives leaving highly segregated neighborhoods. Accordingly, we formulate the following hypothesis: the greater the proportion of non-natives in the neighborhood, the higher the effect of SI among natives.

## Materials and methods

Our analysis is based on longitudinal Swedish register data for Stockholm County (1998–2017). Besides offering precise individual census information on demographic and socio-economic aspects, this unique dataset provided by Statistics Sweden tracks the residential mobility of all residents with almost no missing data. The register data used in this study come from Statistic Sweden and has the ethical approval from the Ethics Review Authority in Sweden (Etikprövningsmyndigheten, Dnr 20137850-31/5). Besides the data was collected in accordance with the EU General Data Protection Regulation. However, given that the share of pruned cases for the subsample of non-natives was very high after the matching procedure, we excluded them from the analysis to avoid misleading, inconclusive results [60].

The spatial residential location of households is measured at the 100m x 100m square-level (henceforth ‘residential area’). Our main outcome, moving-out, is a binary indicating a change in the residential area between two consecutive years. (See S3 Table in S2 File for a robustness analysis on a subsample of households that stay and move only within the region of Stockholm county).

The basis of our analytical strategy involves analyzing the probabilities of moving-out by comparing on the one hand natives exposed to one or more native out-movers and no native in-movers, and on the other hand, natives exposed to no change (i.e. neither in- nor out-movers). The level of granularity offered by the registers thus allows us to capture in a simple way the basic intuition behind our SI hypothesis, whereby having previously observed others moving-out increases the probability of moving-out in comparison to the scenario in which there are neither in- nor out-movers, provided that those exposed to out-movers do not also experience a change in the area due to in-movers in such a manner that could trigger further out-mobility undue to the treatment alone.

Most importantly, this design allows us to adjust for relevant confounders that are likely to modify the effect of SI across individuals and neighborhood areas. In particular, we follow Shalizi and Thomas [61] (translated to the neighborhood context) and argue that the likelihood of following previous out-movers is confounded by factors that account for the high degree of similarity between neighbors within a particular area, which can also cause moving-out behavior. This latter property, generally known as homophily [62], is a well-documented fact in residential segregation, especially along socioeconomic and ethnic lines [5, 22]. Hence, adjusting for factors that account for neighborhood mobility will allow us to differentiate the SI effect from scenarios in which similar movers may show a higher likelihood of moving as a result of some shared attribute.

As described in the previous section, we adjust for four main types of confounding factors that influence residential mobility behavior. First, we include life-course covariates. In particular, we adjust for natives’ age, type of family, civil status, the length of stay [27], and type of tenancy tenure [10]. Second, we include socioeconomic factors related to the individuals and the residential areas, such as the number of years in education, their disposable income (logged), and the median disposable income (logged) of the residential area [9]. Third, we account for ethnic preferences by including natives’ country of birth according to their parents’ country of birth (either both of Swedish origin, otherwise from EU-15/North-America), and the proportion of non-natives in the residential area [32]. Finally, we adjust for the number of inhabitants in the residential area to account for the visibility of out-movers [63].

One potential drawback of our analytical strategy is that the groups to be compared might differ too greatly on a given covariate. Table 1 shows the situation for the Full sample. As can be seen, some individual covariates are somewhat imbalanced across groups, such as the proportion of natives with children, the proportion of renters and owner-occupiers, or the ethnic composition, socioeconomic status, and number of inhabitants in the residential area. Scholars have previously argued that standard regression adjustment alone might be an inefficient way to estimate causal effects as a result of larger standard errors [64] and increased dependence on the functional form presumed to gauge effects [60].

**Table 1:** Descriptives for the entire sample of natives exposed to neither in- nor out-movers, and of natives exposed to at least one person previously having moved-out from their residential area and no in-movers for the county of Stockholm.

Full sample										
Mean			Variance		Median		1Q		3Q	
	No change	Out-movers	No change	Out-movers	No change	Out-movers	No change	Out-movers	No change	Out-movers
*Single*	0.25	0.25	0.19	0.19	-	-	-	-	-	-
*Married*	0.63	0.64	0.23	0.23	-	-	-	-	-	-
*Divorced*	0.12	0.11	0.1	0.1	-	-	-	-	-	-
*With children*	0.55	0.62	0.25	0.24	-	-	-	-	-	-
*No children*	0.29	0.25	0.2	0.19	-	-	-	-	-	-
*Sweden*	0.84	0.83	0.13	0.14	-	-	-	-	-	-
*EU-15/US/Canada*	0.16	0.17	0.13	0.14	-	-	-	-	-	-
*Renter*	0.01	0.03	0.01	0.03	-	-	-	-	-	-
*Owner*	0.01	0.03	0.01	0.03	-	-	-	-	-	-
*Age*	50.58	49.96	254.21	248.09	50	50	39	40	62	61
*Years of education*	12.13	12.4	8.77	8.11	12	12	11	11	14	15
*Disposable income (log)*	7.44	7.48	0.66	0.62	7.44	7.48	7.09	7.14	7.84	7.86
*Length of stay*	6.75	6.97	22.92	23.96	6	6	3	3	10	10
*Neigh*. *pr*. *non-westerners*	0.06	0.09	0.01	0.02	0	0.05	0	0	0.09	0.12
*Neigh*. *median disp*. *income (log)*	7.45	7.47	0.25	0.14	7.45	7.46	7.22	7.27	7.69	7.67
*Neigh*. *N inhabitants*	12.11	24.54	145.18	584.89	10	20	4	13	17	29
*N*	1 300 050	2 977 420								

*Note*: Each row presents the mean, variance, median, 1Q and 3Q value of a covariate, either in terms of its numerical (logged) scale or as a proportion in the case of categorical covariates. Each value is reported for the entire sample prior to matching (Full sample).

To overcome these issues, we apply Coarsened Exact Matching (CEM) [65]. As a matching technique, CEM intends to improve the degree of similarity between groups given a set of covariates while adjusting for them [66]. Moreover, CEM is a non-parametric method, and as such it makes no assumptions on the relationships between covariates and “treatment assignment.” In a nutshell, CEM starts by applying the binning function *H*(*X_j_*), which discretizes—coarses—each covariate *X_j_* separately. This discretization procedure is essential, and it should be done following theoretical arguments when possible [65]. In the next step, the algorithm generates the Cartesian product of all discretized covariates, i.e., *H*(*X*_1_)×…×*H*(*X_j_*), resulting in the multivariate cross-tabulation *H*(***X***). Finally, CEM assumes that observations within each multidimensional cell are equal except for treatment and applies a weight *w* to each observation *i*. Cells with observations belonging only to one group (either treated or control) receive a weight of 0 (i.e., are pruned), while the remaining ones receive the following:

$$w_{i}\left\{ \begin{array}{l} 1,\;i\in T^{S}\\ \frac{m_{C}}{m_{T}}\frac{m_{T}^{S}}{m_{C}^{S}},\;i\in C^{S} \end{array}\right.$$

Where *m_C_* corresponds to the total number of control cases, and $m_{C}^{S}$ to the number of control cases in cell/stratum *S*. Finally, *m_T_* and $m_{T}^{S}$ refer to the treated counterparts.

As can be seen in Table 2, CEM produces a remarkable improvement in the average value of all covariates between the groups, as well as in the other statistics displayed in the Table (see S1 Appendix in S2 File for a complete overview of the performance of CEM).

**Table 2:** Descriptives for the sample of natives exposed to neither in- nor out-movers, and of natives exposed to at least one person previously having moved-out from their residential area and no in-movers for the county of Stockholm after matching.

Matched sample										
Mean			Variance		Median		1Q		3Q	
	No change	Out-movers	No change	Out-movers	No change	Out-movers	No change	Out-movers	No change	Out-movers
*Single*	0.23	0.23	0.18	0.18	-	-	-	-	-	-
*Married*	0.68	0.68	0.22	0.22	-	-	-	-	-	-
*Divorced*	0.09	0.09	0.08	0.08	-	-	-	-	-	-
*With children*	0.64	0.64	0.23	0.23	-	-	-	-	-	-
*No children*	0.26	0.26	0.19	0.19	-	-	-	-	-	-
*Sweden*	0.86	0.86	0.12	0.12	-	-	-	-	-	-
*EU-15/US/Canada*	0.14	0.14	0.12	0.12	-	-	-	-	-	-
*Renter*	0.01	0.01	0.01	0.01	-	-	-	-	-	-
*Owner*	0.02	0.02	0.02	0.02	-	-	-	-	-	-
*Age*	49.85	49.96	250.95	248.81	49	50	40	40	61	61
*Years of education*	12.39	12.4	8.16	8.1	12	12	11	11	15	15
*Disposable income (log)*	7.47	7.48	0.42	0.43	7.45	7.46	7.14	7.14	7.82	7.83
*Length of stay*	6.89	7.03	23.42	24.21	6	6	3	3	10	11
*Neigh*. *pr*. *non-westerners*	0.07	0.07	0.01	0.01	0.04	0.04	0	0	0.1	0.1
*Neigh*. *median disp*. *income (log)*	7.47	7.48	0.12	0.11	7.47	7.47	7.27	7.28	7.66	7.67
*Neigh*. *N inhabitants*	18.58	20.29	152.17	152.18	17	19	10	13	25	26
*N*	1 080 017	2 528 939								

*Note*: Each row presents the mean, variance, median, 1Q and 3Q value of a covariate, either in terms of its numerical (logged) scale or as a proportion in the case of categorical covariates. Each value is reported for the sample after matching (Matched sample). The smaller number of cases in each group after matching is due to pruning. See S1 Appendix in S2 File for a more detailed report on how the matching was performed.

Finally, to ensure that covariates are always measured *before* natives are exposed to out-movers and that the outcome is always measured *after* the exposure [67], we follow Aral et al. [53] and dynamically rematch individuals over the available years. We do so by splitting the longitudinal information available for each individual into overlapping time-segments of three consecutive years (1998-1999-2000, 1999-2000-2001, …, 2015-2016-2017), each of which we call a *matching trial*. The first year of each of these trials determines, for each individual, the value of the covariates, the second the value of the treatment exposure, and the third the outcome: the probability of a household leaving its current residential area. This organization of time guarantees that the relationships between covariates, treatment and outcome remain their causal structure for all the available years, which allows us to fairly match natives for each trial. Most importantly, by applying this dynamic matching we ensure that natives are always matched with other similar natives within any given trial, and also that they are conveniently rematched in the case of any of their covariate values changing over time. Naturally, eligible natives for analysis must have complete information about their covariates, exposure, and mobility as required for any given trial, otherwise they are discarded for that trial.

Once CEM has been applied to each trial, we gauge our SI estimates by applying a weighted linear probability model (LPM). The model consists solely of the binary moving outcome, the variable indicating which group natives are in, and the CEM weights. The choice of this approach rather than the more conventional use of logistic regression for the analysis of binary outcomes is because we are concerned with estimating the difference between groups rather than on predicting, and also with comparing the estimates across non-nested models, which is not feasible using logistic regression without making what are typically very strong and unverifiable assumptions [68]. To gauge the statistical significance of differences between estimates from different models, we follow Clogg, Petkova and Haritou [69].

## Results

### The SI effect on residential out-mobility

**Fig 1:**
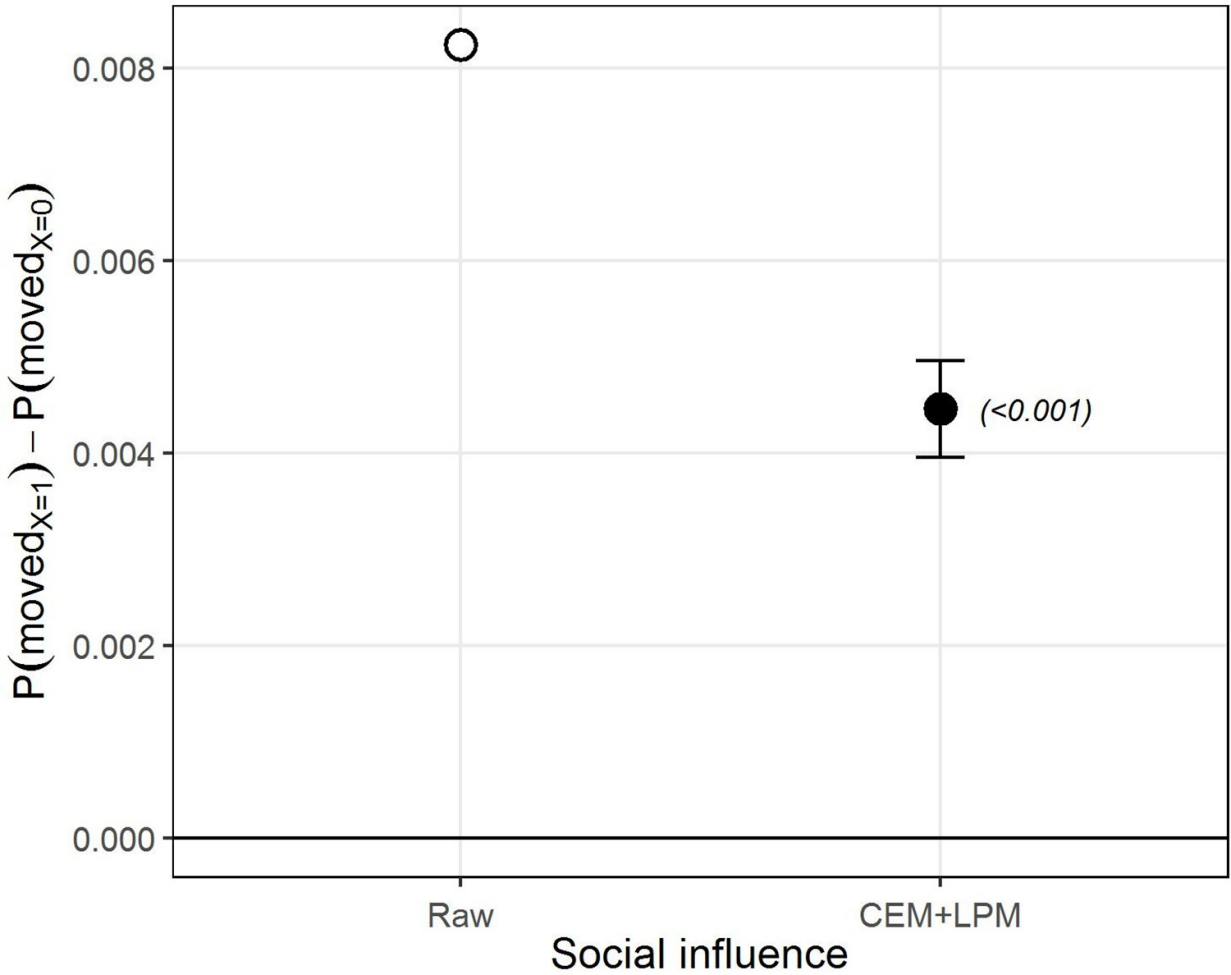
The SI effect on residential out-mobility. Difference in the probability of moving-out between natives exposed to out-movers and no in-movers (X = 1) and natives exposed to no change (neither in- nor out-movers) (X = 0). The estimates are the β coefficient from the LPM on the raw data (in white) and after applying CEM (in black). Error bars indicate 95% confidence intervals, p-value in parenthesis. The straight horizontal line indicates no effect.

Fig 1 presents the results for our first hypothesis: the social influence effect on neighborhood mobility. The figure shows the SI effect after matching (CEM+LPM, black) as captured by the beta coefficient of the LPM. Error bars represent confidence intervals (CI) at the 95%, with p-values in parenthesis (See S1 Table in S2 File for results and statistics in tabulated form). To give a sense of the adjustment made by CEM, we compare it to the estimate made before matching (Raw, white), thus without incorporating any of the covariates.

As can be observed in Fig 1, the model shows a clearly positive estimate for moving-out after being previously exposed to out-movers by comparison with exposure to no change. The same estimate with no adjustment made for any covariates seems to notably overestimate the effect by comparison with the estimate after CEM. The difference between the groups is highly statistically significant (p-value<0.001). At the same time, the estimates for moving-out seem to be rather low, partly due to the much higher predisposition not to move, which has also been noted by other mobility scholars [7, 25]. In addition to factors already noted by those authors, we hypothesize that this is also partly due to the strength with which the SI signal is received by the individuals concerned, which we analyze below.

### The strength of the SI effect on residential out-mobility

While the previous result provides evidence for the effect of SI on residential out-mobility, the following section evaluates how the strength of the signal made by out-movers leaving residential areas is affected in four specific domains: the number of previous out-movers, the density of the residential area, the spatial distance from previous out-movers, and the ethnic composition of the residential area.

**Fig 2:**
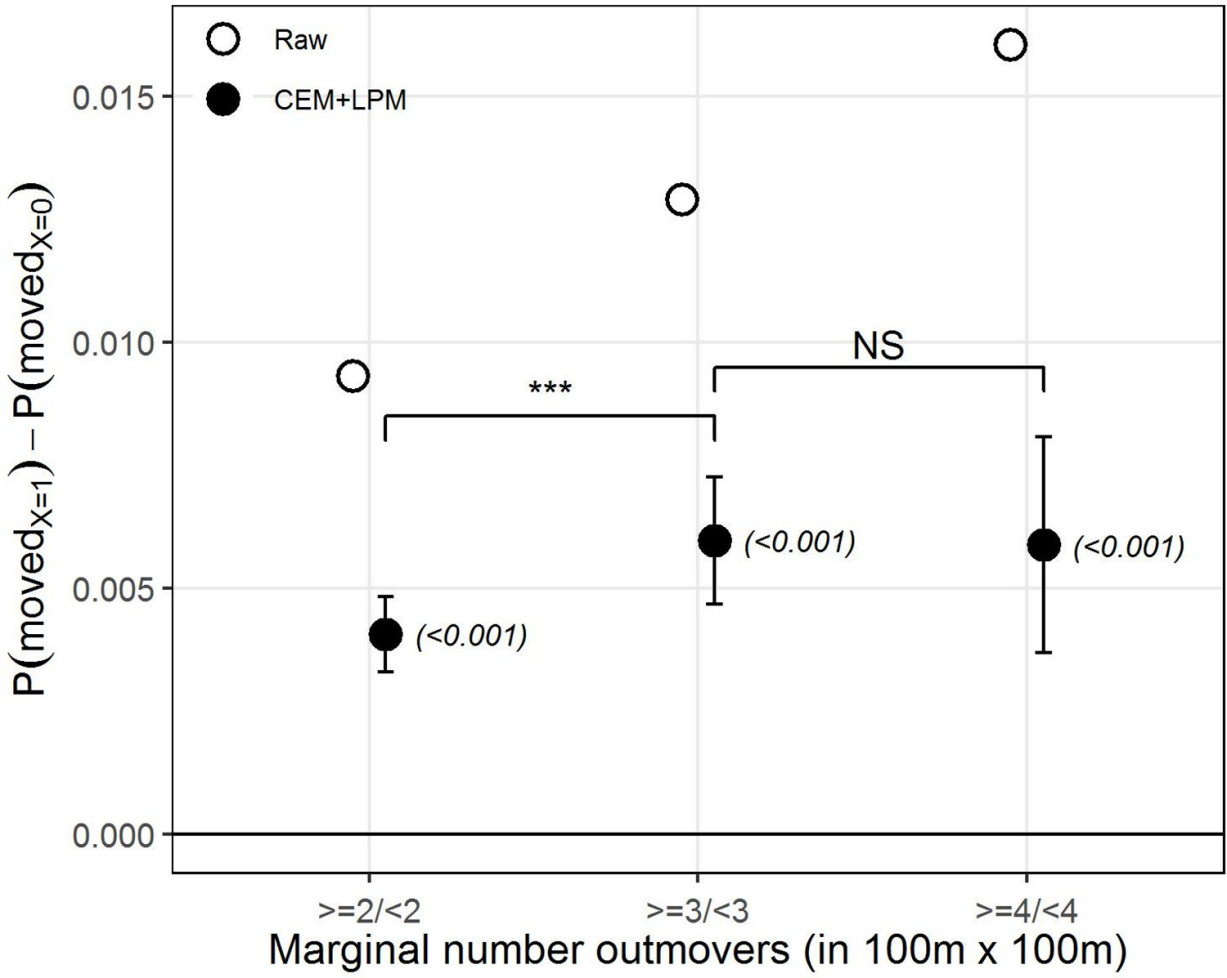
The marginal effect of the number of previous out-movers on the strength of SI. Difference between natives who are exposed only to 2, 3, or 4 or more previous out-movers (X = 1, left), or fewer than this (X = 0, right), respectively. The estimates are the β coefficient from the LPM on the raw data (in white) and after applying CEM (in black). Error bars indicate 95% confidence intervals, p-values in parentheses. The straight horizontal line indicates no effect. The upper bars indicate whether coefficients across models are statistically significantly different from one another. ‘***’ *p-value*<0.001, ‘**’ *p-value*<0.01, ‘*’ *p-value*<0.05, ‘NS’ *p-value*>0.05.

We start by analyzing the marginal effect on the strength of SI by comparing natives who are exposed to a larger (left) or smaller (right) number of previous out-movers, for each binary configuration respectively (see Fig 2). In line with our expectations, the plot clearly shows that being exposed to a larger number of out-movers increases the probability of moving-out compared to a lower exposure, as indicated by the positive sign and high statistical significance of the estimates. The largest increase in this probability seems to occur when the previous number of out-movers increases from 2 to 3, as indicated by the statistically significant difference between the two. An increase of 4 or more out-movers also seems to yield an increase in the probability of moving-out compared to a lower number, although the estimate does not seem to be either qualitatively nor statistically significantly different from an increase of 3 or more out-movers. Again, the model without covariates appears to markedly overestimate the effects by comparison with the estimate after CEM.

**Fig 3:**
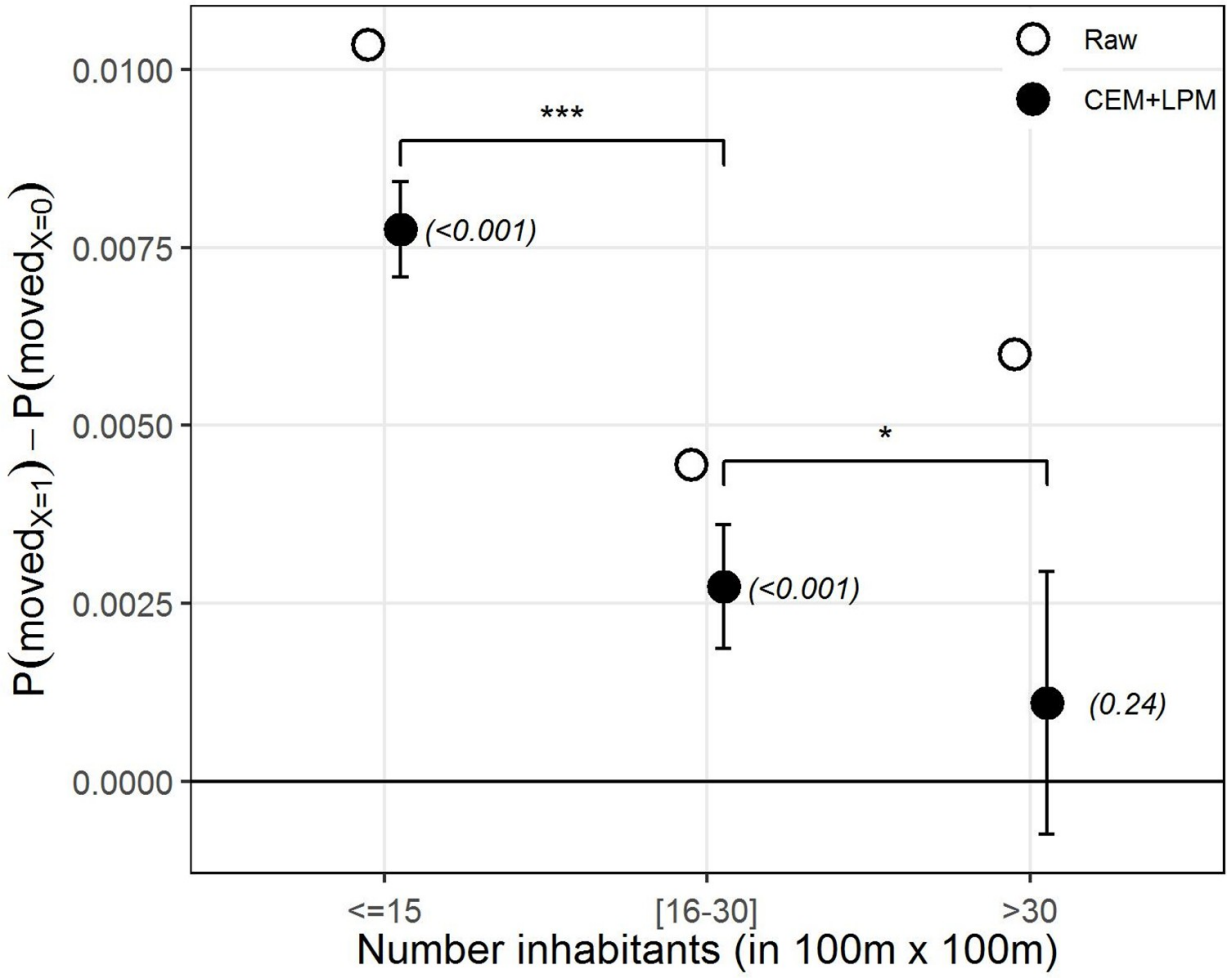
The effect of residential density on the strength of SI. Effect heterogeneity between natives exposed to out-movers (X = 1) and natives exposed to no change (X = 0) in 100m x 100m residential areas with different numbers of inhabitants: (1) less or equal to 15; (2) between 16 and 30; (3) more than 30. The estimates are the β coefficient from the LPM on the raw data (in white) and after applying CEM (in black). Error bars indicate 95% confidence intervals, p-values in parentheses. The straight horizontal line indicates no effect. The upper bars indicate whether coefficients across models are statistically significantly different from one another. ‘***’ *p-value*<0.001, ‘**’ *p-value*<0.01, ‘*’ *p-value*<0.05, ‘NS’ *p-value*>0.05.

We continue by analyzing the effect of SI across residential areas with different total numbers of inhabitants (see Fig 3). As can be seen from the plot, CEM estimates seem to show a downward trend in the effect on moving-out as the area becomes more densely populated. We observe that SI seems to have a marked effect in residential squares with relatively low population density, here defined as those with up to 15 inhabitants. The effect remains positive and statistically significant for areas with a medium number of inhabitants, between 16 and 30, although the effect is clearly weaker than for the lower density case, with the difference between this and the lower density scenario being statistically significant. Finally, the model seems to indicate no effect after crossing the threshold of 30 inhabitants, partly because such cases are much less common in the data. These results are also in line with our expectations, with lower density areas allowing previous out-movers to be more easily noticed than higher density areas. Interestingly, the estimates yielded by CEM show an important correction in relation to higher density areas by comparison with the model without adjustment, which instead shows a larger effect that is most likely capturing out-moving behavior due to other factors in more high-density urban areas.

**Fig 4:**
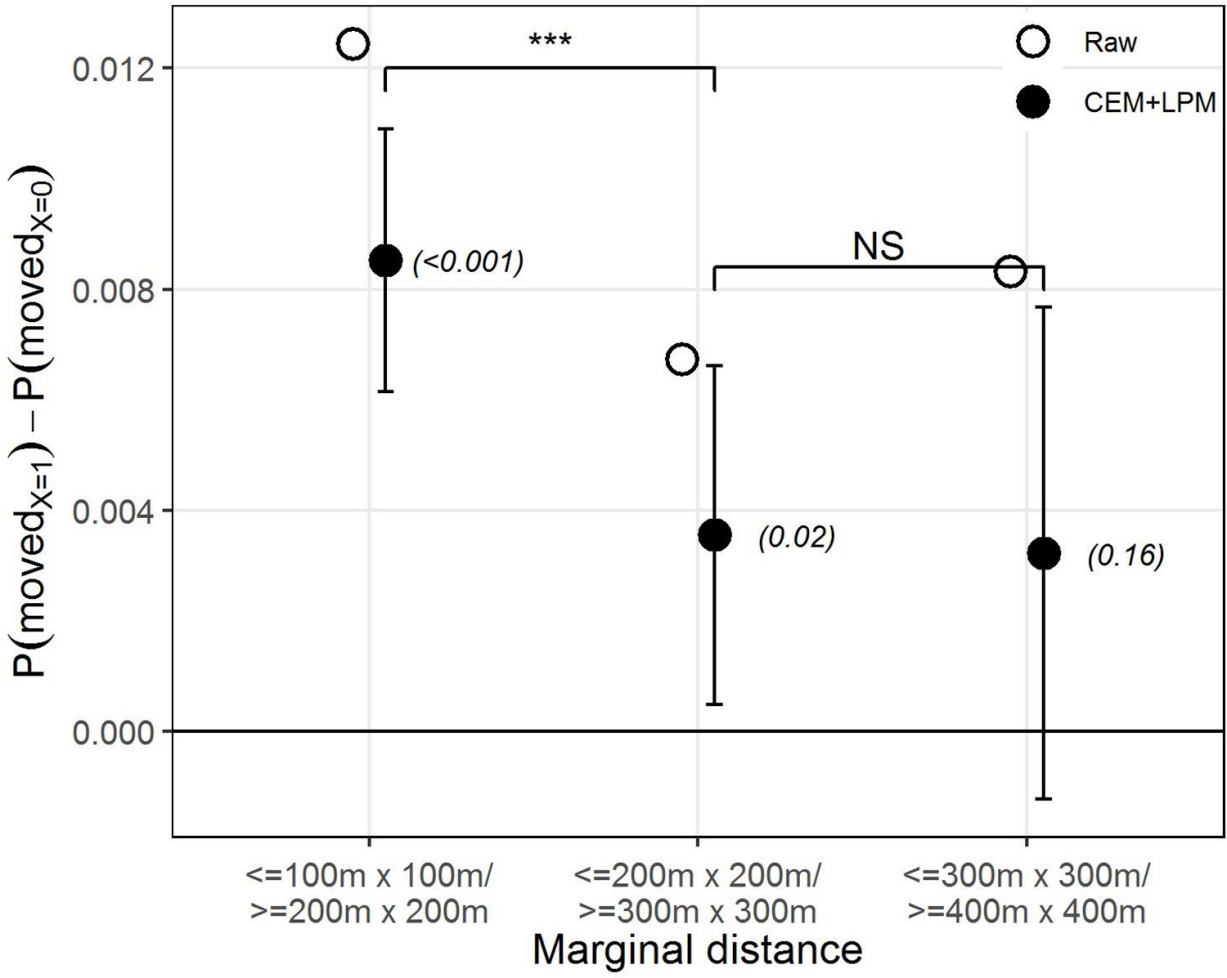
The marginal effect of distance on the strength of SI. Difference between natives being exposed only to out-movers leaving from a point close to their own residential location (X = 1, upper) or from farther away (X = 0, lower), respectively for three different spatial levels: (1) 100m x 100m; (2) 200m x 200m; (3) 300m x 300m. The maximum area is bounded by the 400m x 400m square at all levels. The estimates are the β coefficient from the LPM on the raw data (in white) and after applying CEM (in black). Error bars indicate 95% confidence intervals, p-values in parentheses. The straight horizontal line indicates no effect. The upper bars indicate whether coefficients across models are statistically significantly different from one another. ‘***’ *p-value*<0.001, ‘**’ *p-value*<0.01, ‘*’ *p-value*<0.05, ‘NS’ *p-value*>0.05.

Fig 4 shows the marginal effect on SI of increasing the spatial distance between previous out-movers and the location of natives. To gauge this effect, we compare natives exposed to out-movers that either left from “closer” distances from their residential location (indicated by the upper product in each category) or from “farther away” (the lower product in each category), bounded by the 400m x 400m square. As expected, the plot indicates a greater SI effect when out-movers moved from more proximate areas compared to more distant ones. As can be seen from the Fig 4, the estimates are greatest when the comparison is made between exposure to out-movers either within or beyond an area of 100m x 100m, after which the effect is weak or not statistically significant at all. The 100m model also seems to yield an estimate that is statistically significantly different from the 200m model. Thus, the models indicate the SI effect to be strongest when previous out-movers left from the most proximate area, as indicated by the 100m x 100m residential area, to then diminish rapidly the greater the distance separating ego from previous out-movers, yielding no effect beyond the 300m x 300m residential area.

**Fig 5:**
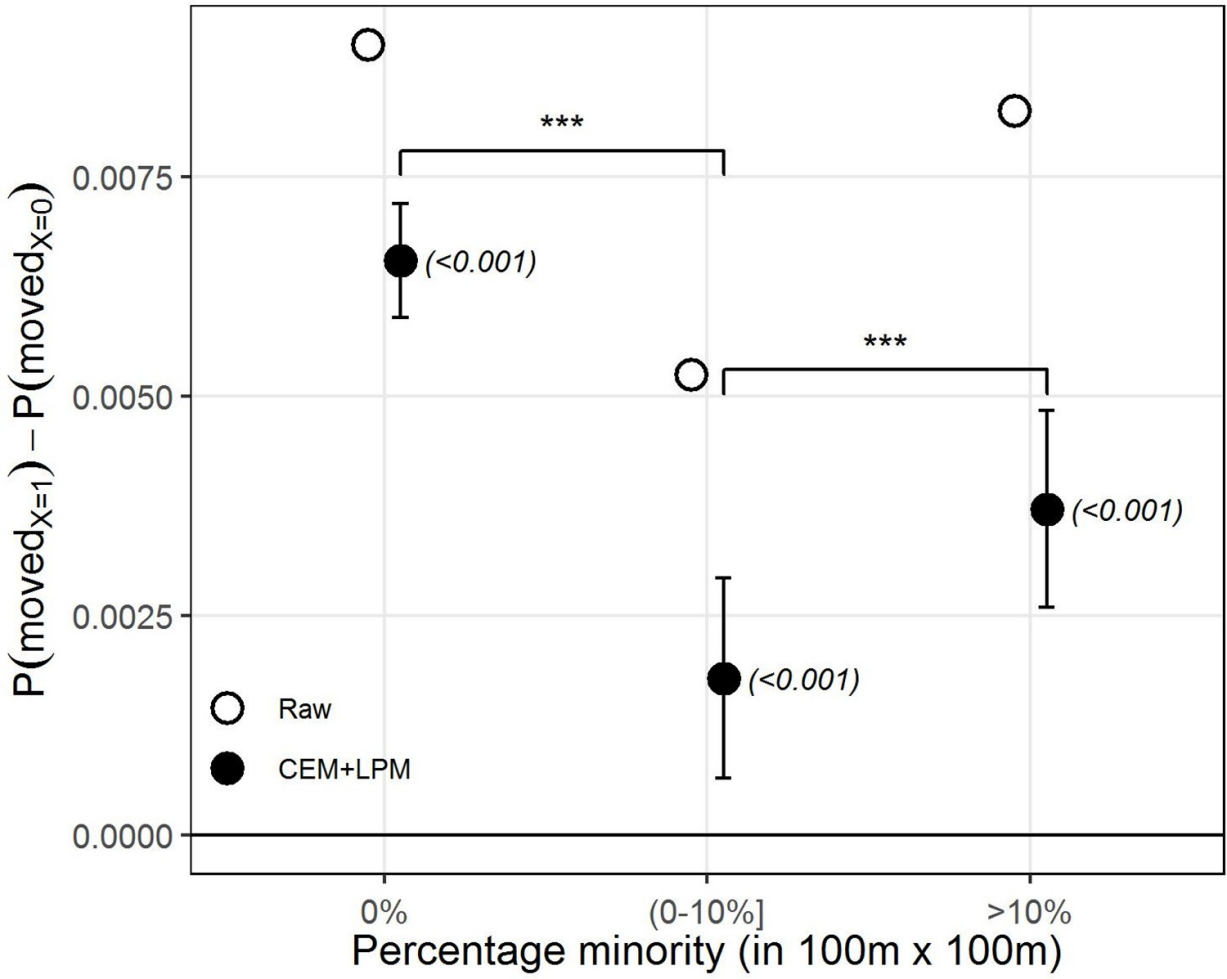
The SI effect across residential areas with varying ethnic compositions. Effect heterogeneity between natives exposed to out-movers (X = 1) and natives exposed to no change (X = 0) in 100m x 100m residential areas across three types of ethnic composition: (1) only natives (left); (2) less than 10% non-natives (middle); and (3) more than 10% non-natives (right). The estimates are the β coefficient from the LPM on the raw data (in white) and after applying CEM (in black). Error bars indicate 95% confidence intervals, p-values in parentheses. The straight horizontal line indicates no effect. The upper bars indicate whether coefficients across models are statistically significantly different from one another. ‘***’ *p-value*<0.001, ‘**’ *p-value*<0.01, ‘*’ *p-value*<0.05, ‘NS’ *p-value*>0.05.

Finally, Fig 5 shows the effect of the ethnic composition of residential areas on the strength of SI. As can be seen from the plot, the estimated effect is clearly positive and statistically significant in areas where the presence of non-natives is null, suggesting evidence for the presence of SI even in native-only areas. Nevertheless, the effect remains positive and statistically significant as areas become more ethnically mixed, although the size of the effect declines, thus leading us to reject the direction of our final hypothesis, that natives moving-out from non-native areas would be more salient—more influential—for other natives than natives leaving native-dominated areas.

Fig 5 highlights two important aspects of the role of SI and ethnic composition on neighborhood mobility. First, it demonstrates that SI processes may partly drive residential mobility independently of other ethnic preference-based explanations, since previous out-movers in native-only areas do not change the ethnic composition by increasing the presence of ethnic minorities. Second, the fact that the SI effect is smaller in ethnically mixed areas might potentially suggest that ethnic preference-based mobility may be stronger than SI in these areas, or that the two processes are entangled in non-linear ways, making it difficult to distinguish them from one another, and potentially making them impossible to identify using only observational data. This indicates a problem that has previously been largely unnoticed in the literature on segregation and residential mobility as a result of having overlooked SI processes of the type defined here, namely that ethnic-based processes may be entangled with SI processes and that the two may be indistinguishable from each other, which at the same time suggests a potential for the study of new mechanisms that combine the role of ethnic preferences and social influence in order to identify new dynamics in residential mobility.

### The SI effect for Göteborg and Malmö

**Fig 6:**
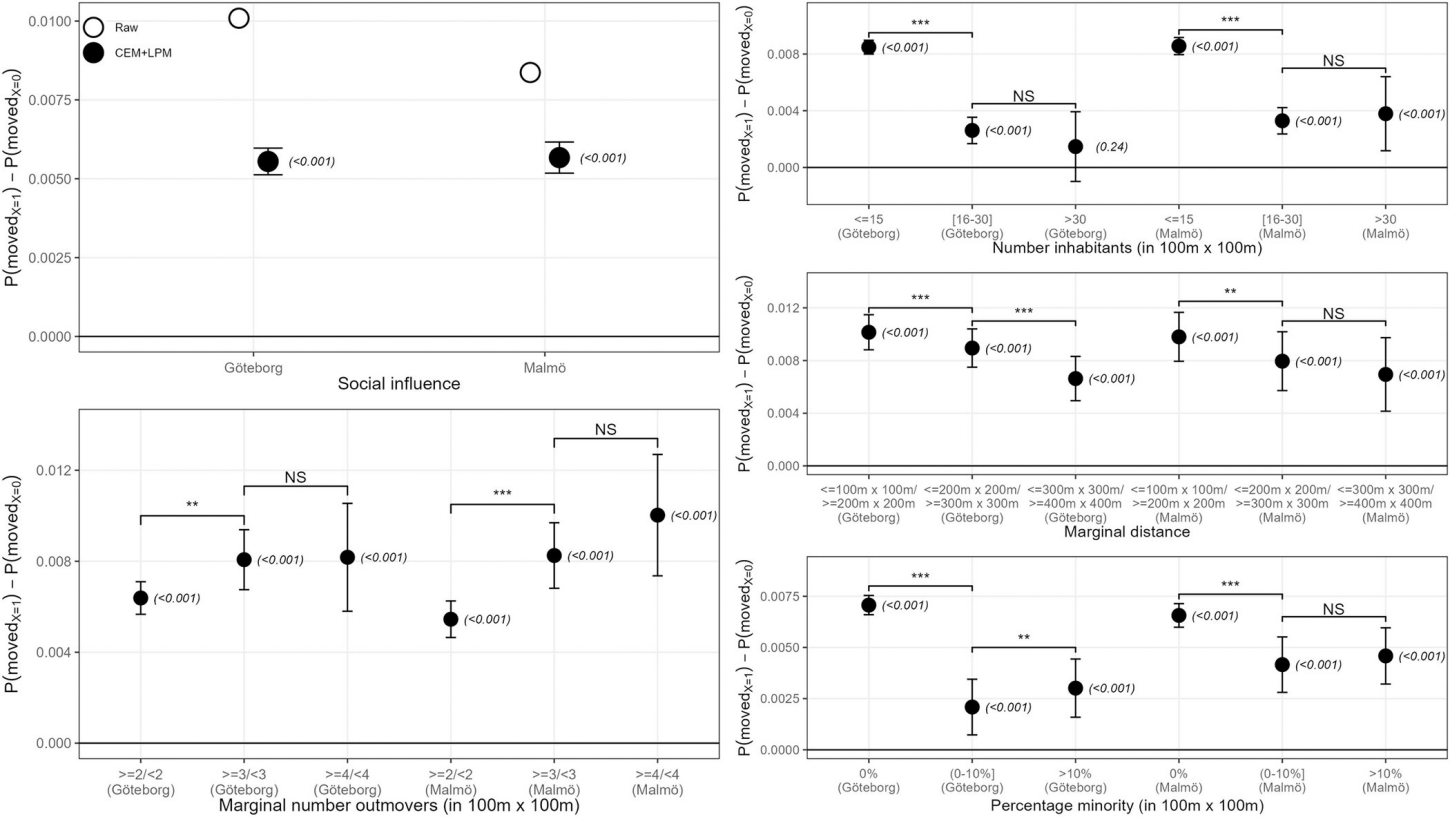
The SI effect and the strength of SI for Göteborg and Malmö. (*Top-left*) Difference in the probability of moving-out between natives exposed to out-movers and no in-movers (X = 1) and natives exposed to no change (neither in- nor out-movers) (X = 0). (*Bottom-left*) Difference between natives who are exposed only to 2, 3, or 4 or more previous out-movers (X = 1, left), or fewer than this (X = 0, right), respectively. (*Top-right*) Effect heterogeneity between natives exposed to out-movers (X = 1) and natives exposed to no change (X = 0) in 100m x 100m residential areas with different numbers of inhabitants: (1) less or equal to 15; (2) between 16 and 30; (3) more than 30. (*Middle-right*) Difference between natives being exposed only to out-movers leaving from a point close to their own residential location (X = 1, upper) or from farther away (X = 0, lower), respectively for three different spatial levels: (1) 100m x 100m; (2) 200m x 200m; (3) 300m x 300m. (*Bottom-right*) Effect heterogeneity between natives exposed to out-movers (X = 1) and natives exposed to no change (X = 0) in 100m x 100m residential areas across three types of ethnic composition: (1) only natives (left); (2) less than 10% non-natives (middle); and (3) more than 10% non-natives (right). The estimates are the β coefficient from the LPM on the raw data (in white, only in Top-left to prevent clutter in the other plots) and after applying CEM (in black). Error bars indicate 95% confidence intervals, p-values in parentheses. The straight horizontal line indicates no effect. The upper bars indicate whether coefficients across models are statistically significantly different from one another. ‘***’ *p-value*<0.001, ‘**’ *p-value*<0.01, ‘*’ *p-value*<0.05, ‘NS’ *p-value*>0.05.

To test our results for other unobservable aspects that might be present in Stockholm as the capital of Sweden but not in other secondary cities (e.g., residential density, the influx of immigrants, job market attractiveness, etc.), we run the same analysis on other cities. Fig 6 shows results of the SI effect and its strength for the second and third most populated Swedish counties, Göteborg and Malmö (see S2 and S3 Tables in S2 File for consulting results in tabular form respectively for each country, as well as S4-S7 Tables in S2 File for descriptive statistics before and after matching, omitted here due to space constraints). As can be seen, the estimates generally follow the same trends found for Stockholm county, although with some differences. For instance, as shown in the top-left plot, the effect of SI is positive and even slightly higher for both Göteborg and Malmö than the one found for Stockholm. Similarly, the values encoding the strength of SI vary accordingly with the results described for Stockholm county, as shown in the rest of the Figure. Concretely, we find that SI strengthens as the previous number of out-movers increases (especially between 2 and 3 out-movers since their difference is statistically significant, although the effect estimated for 4 or more out-movers is also highly statistically significant), and also with greater visibility of out-movers, which we found in (a) small areas with lower population density (with Malmö, unlike Stockholm and Göteborg, showing a positive effect even for small areas with 30 or more inhabitants), as well as in (b) out-movers that lived in close proximity distance from ego before moving out. Finally, both Göteborg and Malmö show a positive SI effect in small areas whose ethnic composition is only composed of natives, whereas, as in the case of Stockholm, the SI effect decreases in ethnically mixed areas, the difference between the types of ethnically mixed areas analyzed being reduced (Göteborg) or not statistically different from one another (Malmö) compared to Stockholm.

### The socio-spatial effect of SI on ethnic residential segregation: A first approximation using an ABM

The analyses presented in this paper have focused on detailing the SI effect on residential out-mobility, with little knowledge about the macro-consequences that this behavior might bring on ethnic residential segregation. In this last section, we evaluate the socio-spatial effect of SI on residential segregation by implementing a stylized agent-based model (ABM).

The simulation is largely based on Schelling’s spatial proximity model [29], and as such, households–belonging to one of two groups–move out of their current neighborhood if the share of same-group neighbors living in their most proximate space descends below 50%. Otherwise, they stay. We extend this model by including the SI effect. This specification follows the same rules that Schelling’s model described before except that now households, closest to the neighbor who has moved out, move depending on the strength of SI (see S1 Appendix in S2 File for consulting the ABM).

**Fig 7:**
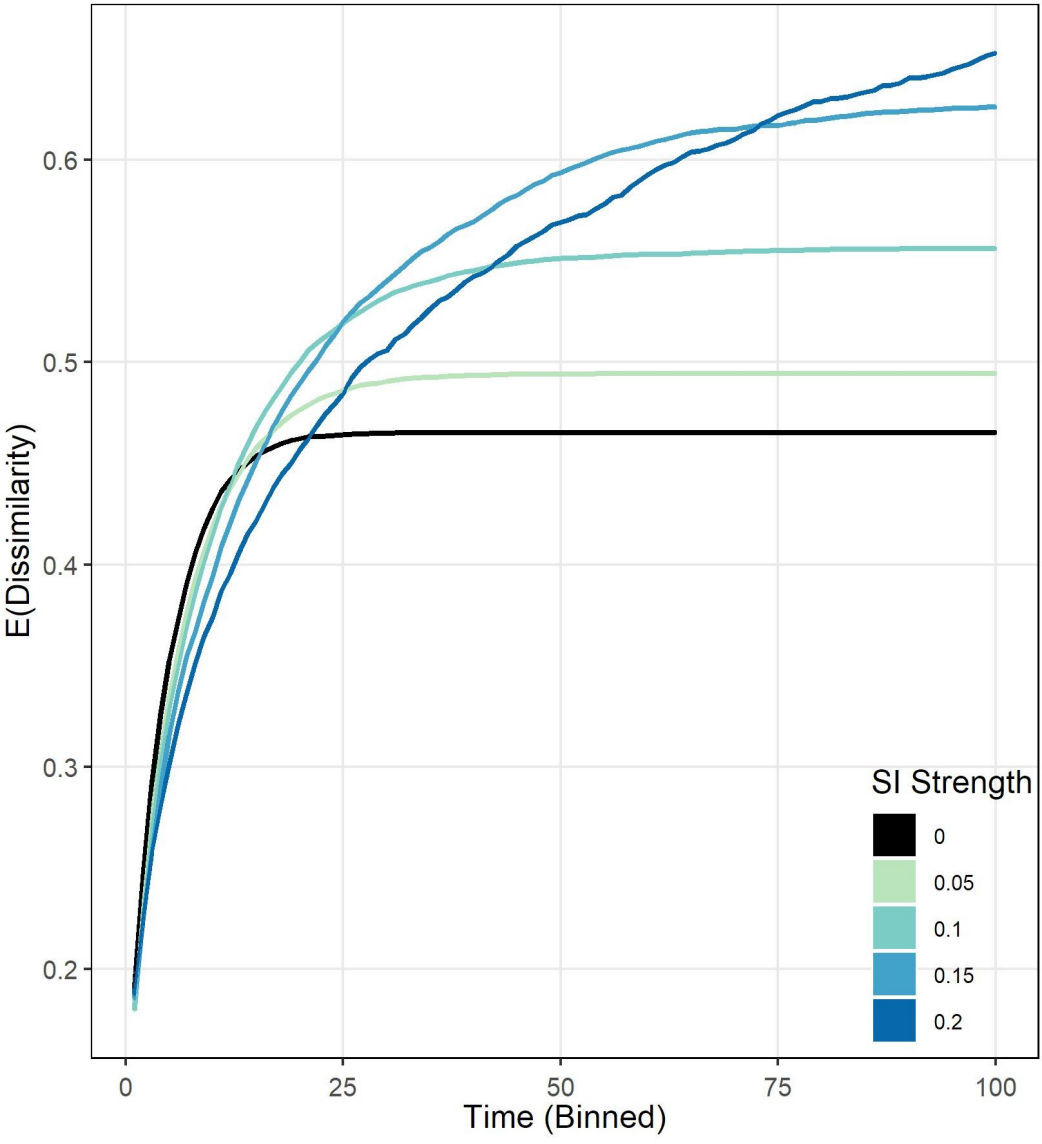
The macro-consequences of SI on ethnic residential segregation. Results from simulations using an agent-based model of Schelling’s (1971) spatial proximity model (SPM) with SI partly determining out-mobility (see main text). Average Dissimilarity Index (Taeuber and Taeuber 1976) for 100 executions for each parameter combination, as a function of time according to different strengths of SI: 0 (no SI, black), 0.05 (lime), 0.1 (turquoise), 0.15 (light blue) and 0.2 (blue). The plots show time binned for clarity (maximum run time is 1e5 for all simulations) and confidence intervals at the 95% level (they are tiny). The black line represents Schelling’s SPM with no SI and it is used as reference. The share of the groups is 50%, and households are randomly distributed at the beginning of the simulation. See the S1 Appendix in S2 File for more information about the ABM.

Fig 7 shows results of simulations for different values of the strength of SI. The plot shows the expected Dissimilarity as a function of time (which is binned to facilitate interpretation) for the scenario without SI (black, referent model) and with SI (non-black colors), whose strength varies from 0.05 to 0.2. As can be seen, the model without SI produces the lowest level of segregation. Interestingly, the plot shows that SI has an ambivalent effect on residential segregation dynamics.

On one side, at the beginning of the simulations, SI *dampens* the level of segregation compared to the model without SI, for which, the greater the strength of SI, the lower the level of segregation achieved at the same stage of the simulation. On the other side, as the simulations continue to unfold, the persistent positive slopes in the SI models produce higher segregation values that actually *surpass* the levels found in the model without SI and continue to increase until much later in the simulation. The greater the strength of SI, the higher the level of segregation achieved at the end of the simulations.

The mechanism behind the ambivalent effect of SI on segregation has to do with how the SI rule interacts with the spatial arrangement of groups over time. Due to agents being randomly allocated on the grid at the beginning of the simulation, SI-driven movements are more likely to not differentiate between any of the two groups. This condition explains why SI exerts a “dampening” effect and reduces segregation at early stages compared to the referent model without SI. Besides, this effect hinders the formation of in-group clusters. However, because of the constant segregation movements produced by Schelling’s model rules, segregation still goes up. As time unfolds and segregation gets more pronounced, we also observe the formation of clusters. Then, the probabilities of moving out for households surrounded by in-group members decrease (they are in clusters), but the ones for households in more undesirable neighborhoods actually increase, bringing with them more households of the same group due to SI which contribute to accelerate the dissolution of mixing areas.

## Conclusion and discussion

SI is a ubiquitous phenomenon in the social sciences. Understanding how SI operates not only provides a better understanding of how individuals take decisions in several areas of their lives, but also provides a first foundation stone for accounting for the development of macro social patterns. The primary goal of this paper has been to make a first exploratory attempt to analyze the presence of a new mechanism, the SI effect, on residential mobility. To achieve this overarching goal, our analysis has focused on answering two main research questions. First, are there SI processes that affect the likelihood of moving-out from a neighborhood after having observed others doing so? Second, is the strength of this effect moderated by the ease with which the signal is perceived?

In order to isolate the effect of SI on moving-out behavior from other alternative explanations found in the literature, we applied CEM and adjusted for several previously tested factors that influence neighborhood mobility. Our results show that after adjusting for theoretically relevant confounders, SI has a significant effect on neighborhood out-mobility and, furthermore, that the strength of this effect is stronger to the visibility of others’ behavior. We measured the visibility of this signal on the following dimensions: signal size, distance to ego, and the signal’s salience given the social environment. Furthermore, our stylized simulation model shows that the effect of SI on segregation dynamics depends on current levels of segregation: when it is low, SI contains the growth of segregation, but when it is high, SI contributes to exacerbate it.

Our work is not exempt from limitations. First, even though we controlled for several factors that the mobility literature has identified as affecting neighborhood mobility, our data cannot rule out the effect of neighborhood reputation on mobility [70]. Second, another important limitation of the registers employed and of our identification strategy is that we cannot adjust for the ethnic composition after natives are exposed to out-movers [60]. This could be problematic because, as has repeatedly been stressed in the segregation literature, natives tend to leave as soon as areas start to become mildly mixed [28]. It is possible that this problem may potentially persist even after adjusting for the ethnic composition of the area prior to the exposure occurring, as the change produced by out-movers might theoretically still push natives to move-out as a result of an increase in the area’s non-native share, rather than as a result of SI per se. This means that SI and ethnic-related motivators for moving-out behavior [22] may be confounded in an essential way, at least in ethnically mixed areas. However, our results provide clear empirical evidence of an effect of SI for the native population with regard to out-mobility. Our simulation approach suggests that SI has an ambivalent effect on segregation dynamics. However, we do not mean to suggest that this is the only way in which SI can be specified. These results must be interpreted in the context of our specific simulation setting.

As regards the size effect of our results, they should be considered in the light of three main characteristics of residential mobility. First, as previous studies have suggested, moving is a highly costly behavior. Evidence shows that only a small fraction of the population moves each year [27, 71]. Thus, given the strong tendency for inertia and for staying in the same neighborhood, finding even small SI effects in this context says something about the strength of SI effects. Second, residential segregation is an inherently dynamic process [29]. As such, careful attention should be paid to small effects that might easily scale-up over time in a non-obvious and unpredictable way [72], as it is the case of reinforced behaviors due to SI dynamics [16]. And third, our model implicitly assumes that all the neighbors are equally able to perceive others’ residential movements. However, even though this perfect information assumption does not hold in real-life situations (even in small areas, there is a chance of not seeing others’ people moving out), we found the SI effect. This means that our results underestimate the effect of SI since there will be situations in which receivers will not receive the signal despite the fact others might move out close to them. Accordingly, our estimations are at the very least conservative of the magnitude of the SI effect.

The individual and social factors surrounding residential mobility decisions make it extremely hard to evaluate the specific contribution of each of them [71]. The challenge becomes even more accentuated due to the heterogeneous configurations of institutional settings affecting those mobility patterns, such as the housing market. To get around the individual–and social–factors, our research design not only adjusts for all of them but is also applied in three different contexts. Regarding the institutional settings, we believe that our results can be extrapolated to other contexts beyond the Swedish housing market. First, our SI effect is focused on out-mobility behaviors, which rules out any constriction due to the existent harsh conditions to find a place in the Swedish market, since everybody is, in principle, free to leave a place. However, one could argue that move-out mobility is conditioned to move-in mobility, such as in the case when households only move out as long as there is an available place to move in. Nevertheless, recent empirical evidence shows that native and non-native populations in Sweden move in similar proportions [73], discarding any major out-mobility restriction and thus being equally susceptible to being influenced in leaving their current neighborhood.

Finally, further research is needed to study the complex ways in which the SI mechanism of neighborhood mobility proposed here interacts with the influential Schelling’s model. It is an open question whether both would reinforce each other or one of them would be more determinant to promote segregation. This line of research could potentially complement and enrich current models of residential segregation that have only taken the effect of households’ neighborhood preferences into account. Another natural extension of this study would be to evaluate the effects of SI on moving-in behavior, in addition to evaluating whether non-native households show similar SI patterns of mobility. Finally, our study also raises some interesting questions in relation to studies of residential segregation. For example, it establishes new factors with which to understand the dynamic aspects of ethnic segregation. This could potentially complement and enrich current models of segregation that have only taken the effect of households’ neighborhood preferences into account.

## Supporting information

S1 File (ZIP)

S2 File (DOCX)
