# Supplementary material for: If you move, I move: The social influence effect on residential mobility
Source: PLoS One. 2022 Jul 6;17(7):e0270783. doi: 10.1371/journal.pone.0270783 (PMC9258896; doi:10.1371/journal.pone.0270783)
Supplement: S2 File — (DOCX) [file pone.0270783.s002.docx]

**S1 APPENDIX**

|  | Difference (Raw) | Difference (CEM+LPM) | P(moved_X=1_\|**Z**) | P(moved_X=0_\|**Z**) | Std. Error | T-value | P-value |
| --- | --- | --- | --- | --- | --- | --- | --- |
| Social influence | 0.161 | 0.087 | 0.056 | 0.052 | 0.005 | 17.5 | <0.01 |
| *Quantity* |  |  |  |  |  |  |  |
| *≥*2/*<*2 | 0.176 | 0.079 | 0.058 | 0.054 | 0.007 | 10.74 | <0.01 |
| *≥*3/*<*3 | 0.235 | 0.114 | 0.061 | 0.055 | 0.012 | 9.56 | <0.01 |
| *≥*4/*<*4 | 0.285 | 0.113 | 0.062 | 0.056 | 0.02 | 5.66 | <0.01 |
| *Density* |  |  |  |  |  |  |  |
| *≤*15 | 0.202 | 0.153 | - | - | 0.008 | 19.01 | <0.01 |
| (15-30] | 0.087 | 0.054 | - | - | 0.007 | 7.39 | <0.01 |
| *>*30 | 0.111 | 0.019 | - | - | 0.008 | 19.01 | 0.13 |
| *Distance* |  |  |  |  |  |  |  |
| *≤*100m x 100m/*≥*200m x 200m | 0.236 | 0.186 | 0.059 | 0.05 | 0.023 | 8.18 | <0.01 |
| *≤*200m x 200m/*≥*300m x 300m | 0.133 | 0.067 | 0.054 | 0.051 | 0.03 | 2.24 | 0.03 |
| *≤*300m x 300m/*≥*400m x 400m | 0.17 | 0.075 | 0.052 | 0.048 | 0.044 | 1.72 | 0.09 |
| *Ethnic composition* |  |  |  |  |  |  |  |
| 0% | 0.178 | 0.128 | - | - | 0.007 | 17.31 | <0.01 |
| (0-10%] | 0.102 | 0.036 | - | - | 0.009 | 3.9 | <0.01 |
| >10% | 0.158 | 0.073 | - | - | 0.01 | 7.41 | <0.01 |
| Covariates included | No | Yes | Yes | Yes | Yes | Yes | Yes |

**S1 Table. Statistical estimates per analytical setting for the county of Stockholm**. The differences between groups are the beta coefficients of the linear probability model applied on the full sample (Raw) and on the matched sample (CEM+LPM) using ordinary-least squares. The models consist of the native out-mobility outcome (binary) for the exposure group being analyzed (also binary). Predicted probabilities, standard errors, t-values and p-values are gauged using the matched sample.

|  | Difference (Raw) | Difference (CEM+LPM) | P(moved_X=1_\|**Z**) | P(moved_X=0_\|**Z**) | Std. Error | T-value | P-value |
| --- | --- | --- | --- | --- | --- | --- | --- |
| Social influence | 0.01 | 0.006 | 0.054 | 0.048 | 2e-04 | 25.73 | <0.01 |
| *Quantity* |  |  |  |  |  |  |  |
| *≥*2/*<*2 | 0.012 | 0.006 | 0.056 | 0.049 | 4e-04 | 17.49 | <0.001 |
| *≥*3/*<*3 | 0.017 | 0.008 | 0.059 | 0.051 | 7e-04 | 11.99 | <0.001 |
| *≥*4/*<*4 | 0.02 | 0.008 | 0.061 | 0.053 | 0.0012 | 6.76 | <0.001 |
| *Density* |  |  |  |  |  |  |  |
| *≤*15 | 0.012 | 0.008 | - | - | 2e-04 | 33.96 | <0.001 |
| (15-30] | 0.005 | 0.003 | - | - | 5e-04 | 5.51 | <0.001 |
| *>*30 | 0.009 | 0.001 | - | - | 0.0013 | 1.17 | 0.24 |
| *Distance* |  |  |  |  |  |  |  |
| *≤*100m x 100m/*≥*200m x 200m | 0.017 | 0.01 | 0.061 | 0.051 | 7e-04 | 14.93 | <0.001 |
| *≤*200m x 200m/*≥*300m x 300m | 0.012 | 0.009 | 0.055 | 0.046 | 7e-04 | 12.05 | <0.001 |
| *≤*300m x 300m/*≥*400m x 400m | 0.011 | 0.007 | 0.054 | 0.047 | 9e-04 | 7.73 | <0.001 |
| *Ethnic composition* |  |  |  |  |  |  |  |
| 0% | 0.011 | 0.007 | - | - | 2e-04 | 29.59 | <0.001 |
| (0-10%] | 0.007 | 0.002 | - | - | 7e-04 | 3.02 | <0.001 |
| >10% | 0.009 | 0.003 | - | - | 7e-04 | 4.16 | <0.001 |
| Covariates included | No | Yes | Yes | Yes | Yes | Yes | Yes |

**S2 Table. Statistical estimates per analytical setting for the county of Göteborg**. The differences between groups are the beta coefficients of the linear probability model applied on the full sample (Raw) and on the matched sample (CEM+LPM) using ordinary-least squares. The models consist of the native out-mobility outcome (binary) for the exposure group being analyzed (also binary). Predicted probabilities, standard errors, t-values and p-values are gauged using the matched sample.

|  | Difference (Raw) | Difference (CEM+LPM) | P(moved_X=1_\|**Z**) | P(moved_X=0_\|**Z**) | Std. Error | T-value | P-value |
| --- | --- | --- | --- | --- | --- | --- | --- |
| Social influence | 0.008 | 0.006 | 0.055 | 0.049 | 3e-04 | 22.53 | <0.001 |
| *Quantity* |  |  |  |  |  |  |  |
| *≥*2/*<*2 | 0.009 | 0.005 | 0.056 | 0.051 | 4e-04 | 13.3 | <0.001 |
| *≥*3/*<*3 | 0.013 | 0.008 | 0.06 | 0.051 | 7e-04 | 11.22 | <0.001 |
| *≥*4/*<*4 | 0.017 | 0.01 | 0.063 | 0.053 | 0.0014 | 7.36 | <0.001 |
| *Density* |  |  |  |  |  |  |  |
| *≤*15 | 0.011 | 0.009 | - | - | 3e-04 | 27.74 | <0.001 |
| (15-30] | 0.006 | 0.003 | - | - | 5e-04 | 6.94 | <0.001 |
| *>*30 | 0.01 | 0.004 | - | - | 0.0013 | 2.84 | <0.001 |
| *Distance* |  |  |  |  |  |  |  |
| *≤*100m x 100m/*≥*200m x 200m | 0.02 | 0.01 | 0.064 | 0.054 | 9e-04 | 10.34 | <0.001 |
| *≤*200m x 200m/*≥*300m x 300m | 0.014 | 0.008 | 0.06 | 0.052 | 0.0011 | 6.97 | <0.001 |
| *≤*300m x 300m/*≥*400m x 400m | 0.012 | 0.007 | 0.06 | 0.053 | 0.0014 | 4.87 | <0.001 |
| *Ethnic composition* |  |  |  |  |  |  |  |
| 0% | 0.009 | 0.007 | - | - | 3e-04 | 22.36 | <0.001 |
| (0-10%] | 0.007 | 0.004 | - | - | 7e-04 | 6.03 | <0.001 |
| >10% | 0.008 | 0.005 | - | - | 7e-04 | 6.54 | <0.001 |
| Covariates included | No | Yes | Yes | Yes | Yes | Yes | Yes |

**S3 Table. Statistical estimates per analytical setting for the county of Malmö**. The differences between groups are the beta coefficients of the linear probability model applied on the full sample (Raw) and on the matched sample (CEM+LPM) using ordinary-least squares. The models consist of the native out-mobility outcome (binary) for the exposure group being analyzed (also binary). Predicted probabilities, standard errors, t-values and p-values are gauged using the matched sample.

*Descriptives for Göteborg*

| Full sample | | | | | | | | | | |
| --- | --- | --- | --- | --- | --- | --- | --- | --- | --- | --- |
| Mean | | | Variance | | Median | | 1Q | | 3Q | |
|  | No change | Out-movers | No change | Out-movers | No change | Out-movers | No change | Out-movers | No change | Out-movers |
| *Single* | 0.27 | 0.24 | 0.2 | 0.19 | - | - | - | - | - | - |
| *Married* | 0.6 | 0.64 | 0.24 | 0.23 | - | - | - | - | - | - |
| *Divorced* | 0.13 | 0.12 | 0.11 | 0.1 | - | - | - | - | - | - |
| *With children* | 0.48 | 0.59 | 0.25 | 0.24 | - | - | - | - | - | - |
| *No children* | 0.32 | 0.27 | 0.22 | 0.2 | - | - | - | - | - | - |
| *Sweden* | 0.9 | 0.89 | 0.09 | 0.1 | - | - | - | - | - | - |
| *EU-15/US/Canada* | 0.1 | 0.11 | 0.09 | 0.1 | - | - | - | - | - | - |
| *Renter* | 0.01 | 0.03 | 0.01 | 0.03 | - | - | - | - | - | - |
| *Owner* | 0 | 0.03 | 0.01 | 0.03 | - | - | - | - | - | - |
| *Age* | 52.03 | 50.7 | 279.62 | 256.78 | 52 | 51 | 40 | 40 | 65 | 62 |
| *Years of education* | 10.91 | 11.43 | 10.78 | 9.78 | 11 | 11 | 9 | 11 | 13 | 14 |
| *Disposable income (log)* | 7.23 | 7.31 | 0.59 | 0.47 | 7.23 | 7.3 | 6.9 | 6.98 | 7.61 | 7.67 |
| *Length of stay* | 7.13 | 7.19 | 24.18 | 25.68 | 6 | 6 | 3 | 3 | 11 | 11 |
| *Neigh. pr. non-westerners* | 0.03 | 0.05 | 0.01 | 0.01 | 0 | 0 | 0 | 0 | 0 | 0.07 |
| *Neigh. median disp. income (log)* | 7.24 | 7.29 | 0.3 | 0.12 | 7.26 | 7.3 | 7.01 | 7.1 | 7.51 | 7.5 |
| *Neigh. N inhabitants* | 8.14 | 18.91 | 81.46 | 229.3 | 5 | 16 | 2 | 10 | 12 | 24 |
| *N* | 1 492 730 | 5 149 132 |  |  |  |  |  |  |  |  |

**S4 Table. Descriptives for the entire sample of natives exposed to neither in- nor out-movers, and of natives exposed to at least one person previously having moved-out from their residential area and no in-movers for the county of Göteborg**. *Note*: Each row presents the mean, variance, median, 1ª and 3Q value of a covariate, either in terms of its numerical (logged) scale or as a proportion in the case of categorical covariates. Each value is reported for the entire sample prior to matching (Full sample).

| Matched sample | | | | | | | | | | |
| --- | --- | --- | --- | --- | --- | --- | --- | --- | --- | --- |
| Mean | | | Variance | | Median | | 1Q | | 3Q | |
|  | No change | Out-movers | No change | Out-movers | No change | Out-movers | No change | Out-movers | No change | Out-movers |
| *Single* | 0.23 | 0.23 | 0.18 | 0.18 | - | - | - | - | - | - |
| *Married* | 0.67 | 0.67 | 0.22 | 0.22 | - | - | - | - | - | - |
| *Divorced* | 0.1 | 0.1 | 0.09 | 0.09 | - | - | - | - | - | - |
| *With children* | 0.6 | 0.6 | 0.24 | 0.24 | - | - | - | - | - | - |
| *No children* | 0.28 | 0.28 | 0.2 | 0.2 | - | - | - | - | - | - |
| *Sweden* | 0.91 | 0.91 | 0.08 | 0.8 | - | - | - | - | - | - |
| *EU-15/US/Canada* | 0.09 | 0.09 | 0.08 | 0.08 | - | - | - | - | - | - |
| *Renter* | 0.01 | 0.01 | 0.01 | 0.01 | - | - | - | - | - | - |
| *Owner* | 0.01 | 0.01 | 0.01 | 0.031 | - | - | - | - | - | - |
| *Age* | 50.75 | 50.81 | 262.71 | 258.53 | 50 | 51 | 40 | 40 | 62 | 62 |
| *Years of education* | 11.31 | 11.32 | 9.87 | 9.78 | 11 | 11 | 11 | 11 | 13 | 13 |
| *Disposable income (log)* | 7.29 | 7.3 | 0.35 | 0.36 | 7.27 | 7.28 | 6.97 | 6.98 | 7.62 | 7.63 |
| *Length of stay* | 7.12 | 7.25 | 24.2 | 24.91 | 6 | 6 | 3 | 3 | 11 | 11 |
| *Neigh. pr. non-westerners* | 0.04 | 0.04 | 0.01 | 0.01 | 0 | 0 | 0 | 0 | 0.05 | 0.05 |
| *Neigh. median disp. income (log)* | 7.28 | 7.29 | 0.13 | 0.11 | 7.29 | 7.29 | 7.08 | 7.09 | 7.49 | 7.49 |
| *Neigh. N inhabitants* | 15.12 | 16.27 | 112.14 | 102.28 | 14 | 15 | 6 | 9 | 21 | 22 |
| *N* | 1 287 737 | 4 589 512 |  |  |  |  |  |  |  |  |

**S5 Table. Descriptives for the sample of natives exposed to neither in- nor out-movers, and of natives exposed to at least one person previously having moved-out from their residential area and no in-movers for the county of Göteborg after matching**. *Note*: Each row presents the mean, variance, median, 1ª and 3Q value of a covariate, either in terms of its numerical (logged) scale or as a proportion in the case of categorical covariates. Each value is reported for the sample after matching (Matched sample). The smaller number of cases in each group after matching is due to pruning.

*Descriptives for Malmö*

| Full sample | | | | | | | | | | |
| --- | --- | --- | --- | --- | --- | --- | --- | --- | --- | --- |
| Mean | | | Variance | | Median | | 1Q | | 3Q | |
|  | No change | Out-movers | No change | Out-movers | No change | Out-movers | No change | Out-movers | No change | Out-movers |
| *Single* | 0.25 | 0.23 | 0.19 | 0.18 | - | - | - | - | - | - |
| *Married* | 0.62 | 0.65 | 0.24 | 0.23 | - | - | - | - | - | - |
| *Divorced* | 0.13 | 0.12 | 0.11 | 0.11 | - | - | - | - | - | - |
| *With children* | 0.47 | 0.56 | 0.25 | 0.25 | - | - | - | - | - | - |
| *No children* | 0.33 | 0.29 | 0.22 | 0.21 | - | - | - | - | - | - |
| *Sweden* | 0.91 | 0.9 | 0.09 | 0.09 | - | - | - | - | - | - |
| *EU-15/US/Canada* | 0.09 | 0.1 | 0.09 | 0.09 | - | - | - | - | - | - |
| *Renter* | 0.01 | 0.02 | 0.01 | 0.02 | - | - | - | - | - | - |
| *Owner* | 0.01 | 0.03 | 0.01 | 0.03 | - | - | - | - | - | - |
| *Age* | 52.1 | 51.1 | 278.15 | 258.77 | 52 | 51 | 40 | 40 | 65 | 62 |
| *Years of education* | 11.2 | 11.52 | 10.82 | 10.05 | 11 | 12 | 9 | 11 | 13 | 14 |
| *Disposable income (log)* | 7.22 | 7.3 | 0.81 | 0.65 | 7.26 | 7.31 | 6.91 | 6.98 | 7.64 | 7.68 |
| *Length of stay* | 7 | 7.12 | 23.8 | 24.35 | 6 | 6 | 3 | 3 | 11 | 11 |
| *Neigh. pr. non-westerners* | 0.05 | 0.06 | 0.01 | 0.01 | 0 | 0 | 0 | 0 | 0.04 | 0.1 |
| *Neigh. median disp. income (log)* | 7.25 | 7.3 | 0.38 | 0.13 | 7.28 | 7.31 | 7.03 | 7.11 | 7.52 | 7.5 |
| *Neigh. N inhabitants* | 9.76 | 20.08 | 95.31 | 309.49 | 7 | 18 | 3 | 11 | 15 | 25 |
| *N* | 3 488 421 | 1 196 499 |  |  |  |  |  |  |  |  |

**S6 Table. Descriptives for the entire sample of natives exposed to neither in- nor out-movers, and of natives exposed to at least one person previously having moved-out from their residential area and no in-movers for the county of Malmö**. *Note*: Each row presents the mean, variance, median, 1ª and 3Q value of a covariate, either in terms of its numerical (logged) scale or as a proportion in the case of categorical covariates. Each value is reported for the entire sample prior to matching (Full sample).

| Matched sample | | | | | | | | | | |
| --- | --- | --- | --- | --- | --- | --- | --- | --- | --- | --- |
| Mean | | | Variance | | Median | | 1Q | | 3Q | |
|  | No change | Out-movers | No change | Out-movers | No change | Out-movers | No change | Out-movers | No change | Out-movers |
| *Single* | 0.21 | 0.21 | 0.17 | 0.17 | - | - | - | - | - | - |
| *Married* | 0.68 | 0.68 | 0.22 | 0.22 | - | - | - | - | - | - |
| *Divorced* | 0.11 | 0.11 | 0.1 | 0.1 | - | - | - | - | - | - |
| *With children* | 0.57 | 0.57 | 0.24 | 0.24 | - | - | - | - | - | - |
| *No children* | 0.3 | 0.3 | 0.21 | 0.21 | - | - | - | - | - | - |
| *Sweden* | 0.93 | 0.92 | 0.07 | 0.07 | - | - | - | - | - | - |
| *EU-15/US/Canada* | 0.07 | 0.08 | 0.07 | 0.07 | - | - | - | - | - | - |
| *Renter* | 0.01 | 0.01 | 0.01 | 0.01 | - | - | - | - | - | - |
| *Owner* | 0.02 | 0.02 | 0.02 | 0.02 | - | - | - | - | - | - |
| *Age* | 51.22 | 51.26 | 265.36 | 261.43 | 51 | 51 | 40 | 41 | 63 | 63 |
| *Years of education* | 11.4 | 11.4 | 9.99 | 10.03 | 11 | 11 | 11 | 11 | 14 | 14 |
| *Disposable income (log)* | 7.28 | 7.29 | 0.42 | 0.43 | 7.27 | 7.29 | 6.97 | 6.98 | 7.63 | 7.64 |
| *Length of stay* | 7.02 | 7.18 | 23.9 | 24.59 | 6 | 6 | 3 | 3 | 11 | 11 |
| *Neigh. pr. non-westerners* | 0.05 | 0.05 | 0.01 | 0.01 | 0 | 0 | 0 | 0 | 0.07 | 0.07 |
| *Neigh. median disp. income (log)* | 7.28 | 7.29 | 0.14 | 0.12 | 7.3 | 7.3 | 7.1 | 7.1 | 7.49 | 7.49 |
| *Neigh. N inhabitants* | 16.3 | 17.33 | 98.18 | 88.97 | 16 | 17 | 9 | 11 | 22 | 23 |
| *N* | 2 965 950 | 1 009 778 |  |  |  |  |  |  |  |  |

**S7 Table. Descriptives for the sample of natives exposed to neither in- nor out-movers, and of natives exposed to at least one person previously having moved-out from their residential area and no in-movers for the county of Malmö after matching**. *Note*: Each row presents the mean, variance, median, 1ª and 3Q value of a covariate, either in terms of its numerical (logged) scale or as a proportion in the case of categorical covariates. Each value is reported for the sample after matching (Matched sample). The smaller number of cases in each group after matching is due to pruning.

*Coarsened Exact Matching*

Our identification strategy takes advantage of the high number of observations available and embrace a matching approach to approximate an “as-if” randomization analysis [1]. Following Stuart [2], we use matching to adjust for confounders and improve the balance between “treatment groups” on the observed covariates (see main text). The improvement in balance ensures that the probability of receiving treatment or control is non-zero for all individuals in any strata of the covariates. In case some strata shows only observations from either the “treated” group or the “control” group, matching prunes those. This procedure ensures positivity and can markedly reduce the uncertainty of a causal estimate by on the one hand decreasing its standard error [3], and on the other hand, by decreasing the bias inherited of the functional relationship presumed to gauge the effect [4].

The method we use is Coarsened Exact Matching (CEM). CEM is a nonparametric matching method from the family of Monotonic Imbalance Bounding methods (MIB) [5]. This family generalizes from the so-called Equal Percent Bias Reducing (EPBR) methods introduced by Rubin. Rather than attempting to reduce the expected imbalance between treatment groups, the goal is to reduce actual in-sample imbalance. This is done by reducing imbalance between treatments groups of each covariate separately and within different regions of the covariate chosen ex-ante by the scientist, rather than reducing overall expected imbalance regardless of confounders strata. This class of methods guarantee that an addition of one variable or a change in their ex-ante coarsed values does not alter the maximal imbalance on the remaining variables. Another way of seeing this is by contrasting both methods to their corresponding experimental designs: while traditional EPBR methods try to emulate a randomized controlled trial, MIB methods follow a blocked-experimental design, where values of the treatment variable are randomly assigned within strata. To apply CEM for the analyses, we used the ‘cem’ package in R [6].

*Bins used for CEM*

The bins used to discretize each covariate for CEM are shown as follows:

- Civil status (qualitative): (1) single; (2) married and registered partners; (3) widow, divorced, or other.
- Family type (qualitative): (1) children under/above 18; (2) no children; (3) other.
- Tenure type (qualitative): (1) renter; (2) owner/cooperative; (3) non-residential.
- Ethnicity (qualitative): (1) Swedish; (2) EU-15/US/Canada.
- Age (numeric): (1) 18-26; (2) 27-35; (3) 36-41; (4) 42-56; (5) 57-112.
- Years of education (numeric): (1) 5-12; (2) 13-20.
- Disposable income, logged (numeric): taking into account the deciles, different for every year.
- Length of stay (numeric): (1) 1-7, (2) 8-18.
- Proportion of non-westerners in area (numeric): taking into account the deciles, different for every year.
- Median disposable income in area (numeric): taking into account the quintiles, different for every year.
- Number of individuals living in each area (numeric): taking into account the deciles, different for every year.

*CEM performance*

As recommended by Stuart [2], any matching implementation should be followed by diagnostics indicating its performance, usually around the improvement in balance. We focus on three measures: (1) the standardized difference in means/proportions before and after matching for each covariate; (2) the so-called $\mathcal{L}_{1}$ measure for the overall multihistogram; (3) the proportion of treated/controls pruned. The first one allows to look at the balance improvement separately for each covariate, and in case of qualitative covariates, to each category. This is by far the diagnostic most relied upon to observe balance improvement, and several recommendations have been made. For instance, Stuart suggests that a good-enough balance results in a standardized difference in means for each covariate below 0.2. We follow her and set 0.2 as the maximal upper bound of maximal imbalance allowed.

Despite being the diagnostic most used, the difference in means by definition overlooks how groups are balanced given the entire distribution of each covariate. To solve this, another advantage of actually using CEM is the availability of the so-called $\mathcal{L}_{1}$ measure, which is defined as:

$$\mathcal{L}_{1}\left( f,g \right)=\frac{1}{2}\sum_{l_{1}\cdots l_{j}\in H(\mathbf{X})} |f_{l_{1}\cdots l_{j}}-g_{l_{1}\cdots l_{j}}|$$

Where $f_{l_{1}\ldots l_{j}}$ are the relative frequencies of observations belonging to the cell with coordinates $l_{1}\ldots l_{j}$ of the multivariate cross-tabulation of the treated units, and $g_{l_{1}\ldots l_{j}}$ the same for control units. $\mathcal{L}_{1}$ outputs values between 0 and 1, with 0 indicating complete overlap between the distributions of both groups (i.e., perfect balance) and 1 otherwise. According to Iacus et al. [7], any real improvement in balance should be indicated by $\mathcal{L}_{1}\left( f^{m},g^{m} \right)\ll\mathcal{L}_{1}\left( f,g \right)$, where $f^{m},g^{m}$ indicate the relative frequency distributions of treated and control after matching. Finally, our third measure takes into account the price for the balance improvement, in terms of the proportion of pruned necessary to reduce imbalance. For instance, too much pruning can make the matched sample too different from the original population, which might be undesirable for the researcher. Nevertheless, a clear advantage of the register data is that the large pool of observations available allows us to prune considerably while still remaining a great bunch of observations, and thus without substantially changing the external validity of the estimates.

Fig in S1 Fig shows each of these measures per analytical setting (see main text), one measure per row. The first row shows the standardized difference in means/proportions for each numerical covariate/category before the matching and after. The straight horizontal line is on 0.1, a conservative level stipulated by Austin [8]. Each covariate is repeated for each of the three consecutive years within which we apply the dynamic matching (see main text). To begin, the plot shows how some covariates are greatly imbalanced before applying CEM, which makes matching a necessary step. The decreased lines for each covariate show that current CEM bins work well enough to get a standardized difference below 0.1 across most covariates, years, and analytical setting. Although some covariates seem to surpass this threshold for some of the years, they still remain below the threshold of 0.2 recommended by Stuart.

**
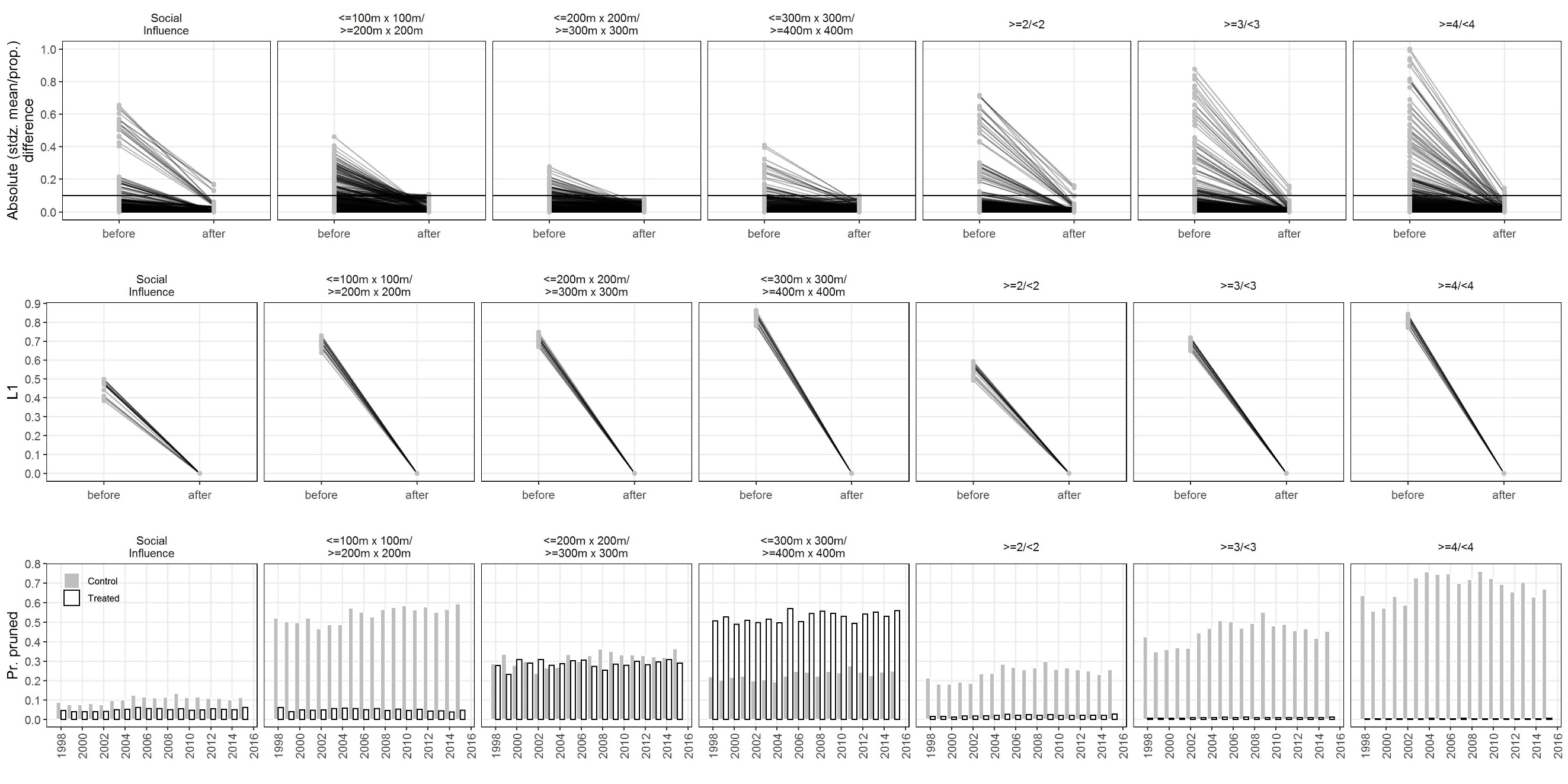
**

**S1 Fig. Performance of CEM as measured by three different measures for the county of Stockholm, each one filling a row.** *(TOP)* Standardized difference in means for numerical covariates, or difference in proportions for each category in qualitative covariates. Each value is shown before and after CEM, linked by a straight line, and repeated for each year where matching is applied. *(MIDDLE)* $\mathcal{L}_{1}$ measure before and after CEM. *(BOTTOM)* Proportion of cases pruned, for the treated group (empty, black-enveloped bars) and the control group (grey, empty-enveloped bars). Performance is checked per analytical setting and three-year trial, displaying the year corresponding to when covariates are measured (i.e., the first one in each trial).

The second row shows measures of the $\mathcal{L}_{1}$ also before and after matching. Because this is a measure that takes the entire histogram into account, there is one measure per each three-years trial. As can be seen, the reduction of imbalance is rather remarkable, as Iacus et al. recommend it should be. Although there is no direct threshold to compare, all the $\mathcal{L}_{1}$ values after CEM are very close to zero, indicating that the multihistogram between groups is highly similar for all groups.

Finally, the last row shows the proportion of each “treated/control” cases pruned, which varies across settings (see main text). As before, results are offered by each three-years trial, displaying the year when the covariates are measured. As can be seen, there is some pruning going on, which heightens almost up to 50% for most cases, and up to 70% in the setting where natives are either exposed to 4 or more out-movers vs less. For each analytical setting, the cases that are mostly pruned are those belonging to the most numerous one, which means that the level of pruning is adequate to reduce imbalance, and at the same time is not too drastic so that it goes in detriment to the external validity of the estimates.

In conclusion, the diagnostic measures discussed show an improvement in “treatment” groups on the observed covariates, which is to a great extent granted by the census information available offered by the Swedish register data.

In addition, the next Figures show the same diagnostics for the counties of Göteborg (Fig in S2 Fig) and Malmö (Fig in S3 Fig). As can be seen, the performance of CEM is also high for Göteborg and Malmö, in a similar way as it is for Stockholm.

**
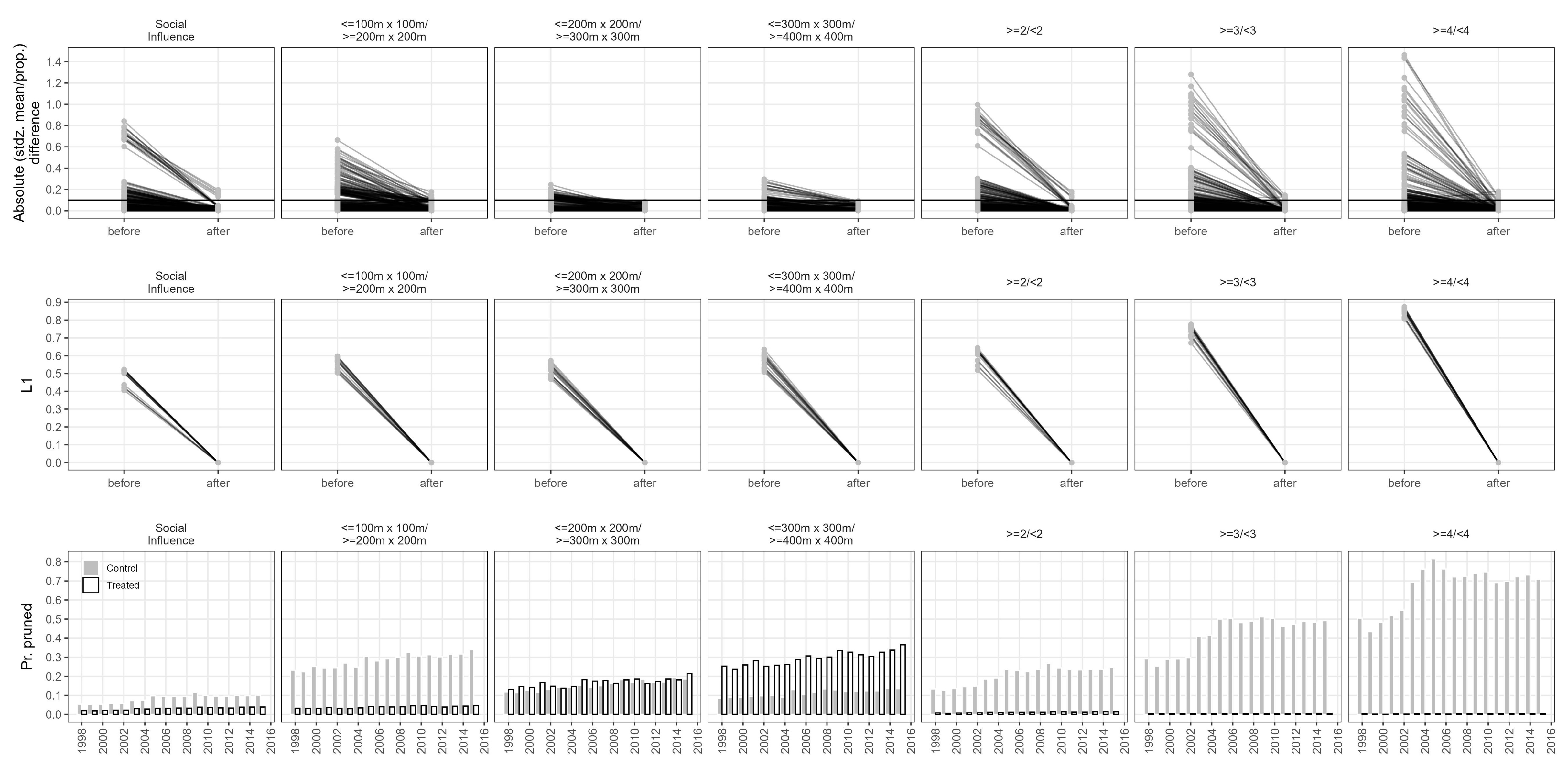
S2 Fig. Performance of CEM as measured by three different measures for the county of Göteborg, each one filling a row**. *(TOP)* Standardized difference in means for numerical covariates, or difference in proportions for each category in qualitative covariates. Each value is shown before and after CEM, linked by a straight line, and repeated for each year where matching is applied. *(MIDDLE)* $\mathcal{L}_{1}$ measure before and after CEM. *(BOTTOM)* Proportion of cases pruned, for the treated group (empty, black-enveloped bars) and the control group (grey, empty-enveloped bars). Performance is checked per analytical setting and three-year trial, displaying the year corresponding to when covariates are measured (i.e., the first one in each trial).

**
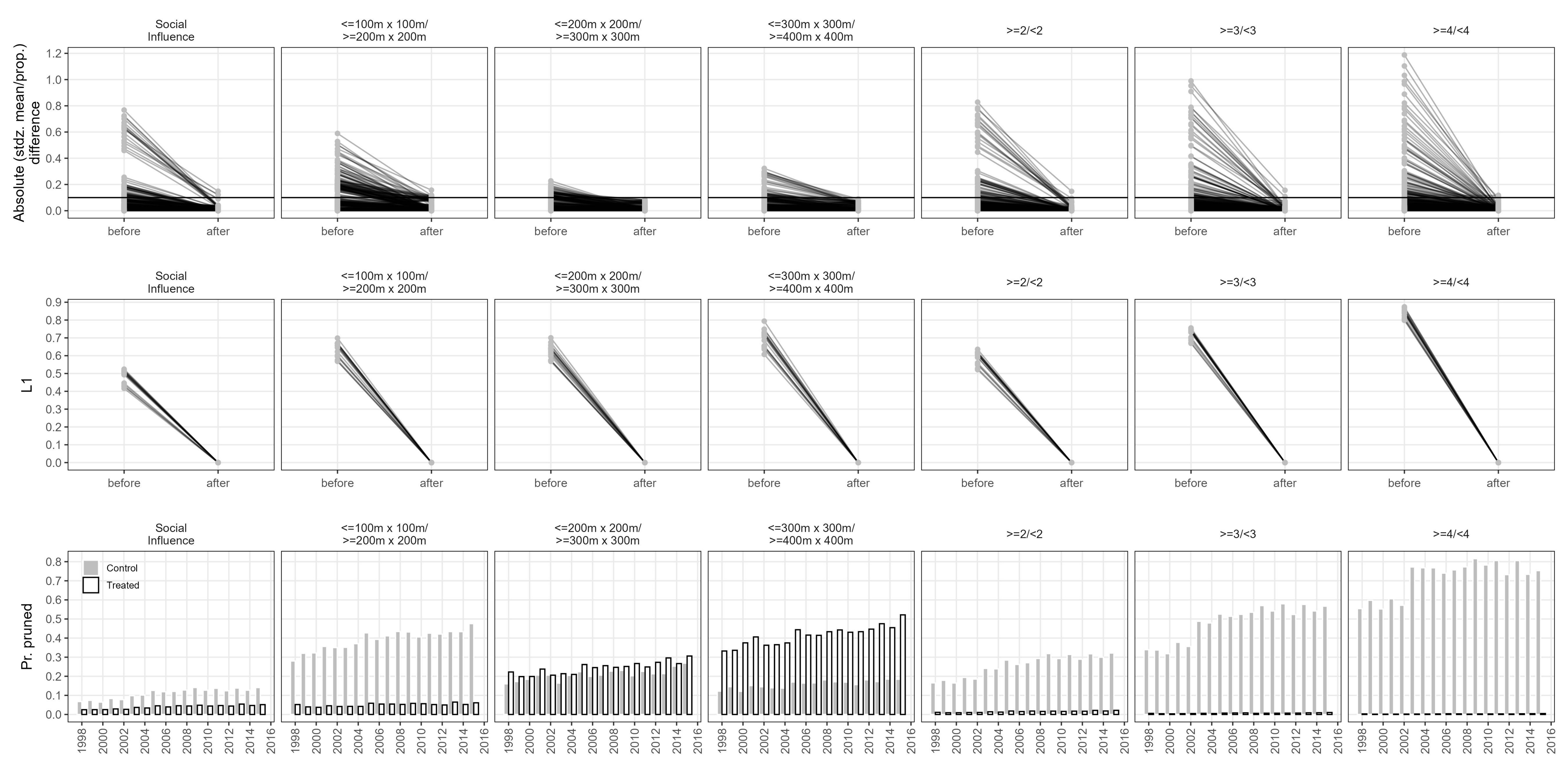
**

**S3 Fig. Performance of CEM as measured by three different measures for the county of Malmö, each one filling a row**. *(TOP)* Standardized difference in means for numerical covariates, or difference in proportions for each category in qualitative covariates. Each value is shown before and after CEM, linked by a straight line, and repeated for each year where matching is applied. *(MIDDLE)* $\mathcal{L}_{1}$ measure before and after CEM. *(BOTTOM)* Proportion of cases pruned, for the treated group (empty, black-enveloped bars) and the control group (grey, empty-enveloped bars). Performance is checked per analytical setting and three-year trial, displaying the year corresponding to when covariates are measured (i.e., the first one in each trial).

*Robustness checks: Family type*

A potential problem with our analyses is that our setting may also capture other processes related with the family structure that follow the same behavioral pattern studied here but which not obey to social influence dynamics. Divorced couples that move at different rates from a common dwelling, or siblings moving out one after another as they enter the university, for instance, are processes that involve being exposed to a co-ethnic leaving, although not a neighbor, and leaving thereafter.

In the paper, family type is a covariate we adjust for, which means that we are already partly dealing with this issue. The problem is that we cannot adjust for changes in the family type at the moment when we measure treatment assignment. Due to the yearly granularity of the recorded data in the registers, we cannot disentangle whether the observed change in the family structure occurred before or after treatment assignment. Thus, in the main text we follow standard procedures in causal inference and avoid adjusting for post-treatment variables to estimate the effect of treatment [4,9].

Despite this, it might still be necessary to re-do the analyses and focus on a subsample of natives who remain their family type *unchanged* during the three-year period used for the analyses. Focusing on Stockholm county, Table in S8 Table shows the results of these robustness checks analyses using the same structure as Table in S1 Table. As can be seen, the estimates reduce their size, but their substantive direction and standard errors remain largely the same.

|  | Difference (Raw) | Difference (CEM+LPM) | P(moved_X=1_\|**Z**) | P(moved_X=0_\|**Z**) | Std. Error | T-value | P-value |
| --- | --- | --- | --- | --- | --- | --- | --- |
| Social influence | 0.004 | 0.003 | 0.033 | 0.03 | 0.0002 | 11.73 | <0.001 |
| *Quantity* |  |  |  |  |  |  |  |
| *≥*2/*<*2 | 0.006 | 0.003 | 0.034 | 0.031 | 0.0003 | 8.18 | <0.001 |
| *≥*3/*<*3 | 0.010 | 0.006 | 0.037 | 0.032 | 0.0006 | 9.97 | <0.001 |
| *≥*4/*<*4 | 0.014 | 0.006 | 0.039 | 0.033 | 0.001 | 6.48 | <0.001 |
| *Density* |  |  |  |  |  |  |  |
| *≤*15 | 0.005 | 0.005 | - | - | 0.0003 | 16.78 | <0.001 |
| (15-30] | 0.003 | 0.002 | - | - | 0.0004 | 4.85 | <0.001 |
| *>*30 | 0.004 | 0 | - | - | 0.0008 | -0.31 | 0.75 |
| *Distance* |  |  |  |  |  |  |  |
| *≤*100m x 100m/*≥*200m x 200m | 0.010 | 0.006 | 0.033 | 0.028 | 0.001 | 5.46 | <0.001 |
| *≤*200m x 200m/*≥*300m x 300m | 0.004 | 0.003 | 0.031 | 0.029 | 0.0013 | 2.09 | 0.04 |
| *≤*300m x 300m/*≥*400m x 400m | 0.005 | -0.001 | 0.027 | 0.027 | 0.0017 | -0.3 | 0.77 |
| *Ethnic composition* |  |  |  |  |  |  |  |
| 0% | 0.004 | 0.004 | - | - | 0.0003 | 14.82 | <0.001 |
| (0-10%] | 0.003 | 0 | - | - | 0.0005 | 0.73 | 0.46 |
| >10% | 0.005 | 0.002 | - | - | 0.0005 | 4.74 | <0.001 |
| Covariates included | No | Yes | Yes | Yes | Yes | Yes | Yes |

**S8 Table.** **Statistical estimates per analytical setting on the subsample of natives remaining family type unchanged in period of analysis for Stockholm county**. *Note*: Estimates on the difference scale are computed both on the full sample (Raw) and on the matched sample (CEM-LPM). Predicted probabilities, standard errors, t-values and p-values are gauged using the matched sample.

*Robustness checks: Mobility within Stockholm county*

The analyses that we show in the main manuscript include households who moved out outside the region of Stockholm county. This opens the possibility that the reasons that motivate the act of moving are others than the covariates we adjust for and the social-influence treatment exposure, such as a change of workplace. The analyses in the main manuscript do not treat this in advance because this involves an adjustment of the outcome variable that could “open” some unobserved causal path through the observed covariates that we adjust for that could bias the estimation of the analyses. Similarly as before, we proceed on repeating the analyses here as a robustness check.

Table in S9 Table shows the same estimates as in the previous tables for those households who stayed and moved within Stockholm County. As can be seen, the quality of the estimates and their statistical significance remain largely the same as those shown and discussed in the main manuscript (see Table in S1 Table).

|  | Difference (Raw) | Difference (CEM+LPM) | P(moved_X=1_\|**Z**) | P(moved_X=0_\|**Z**) | Std. Error | T-value | P-value |
| --- | --- | --- | --- | --- | --- | --- | --- |
| Social influence | 0.008 | 0.004 | 0.056 | 0.052 | 0.0003 | 17.31 | <0.001 |
| *Quantity* |  |  |  |  |  |  |  |
| *≥*2/*<*2 | 0.009 | 0.004 | 0.058 | 0.054 | 0.0004 | 10.41 | <0.001 |
| *≥*3/*<*3 | 0.013 | 0.006 | 0.061 | 0.055 | 0.0007 | 9.04 | <0.001 |
| *≥*4/*<*4 | 0.016 | 0.006 | 0.062 | 0.056 | 0.0011 | 5.24 | <0.001 |
| *Density* |  |  |  |  |  |  |  |
| *≤*15 | 0.010 | 0.008 | - | - | 0.0003 | 22.71 | <0.001 |
| (15-30] | 0.004 | 0.003 | - | - | 0.0004 | 6.17 | <0.001 |
| *>*30 | 0.006 | 0.001 | - | - | 0.0009 | 1.17 | 0.24 |
| *Distance* |  |  |  |  |  |  |  |
| *≤*100m x 100m/*≥*200m x 200m | 0.012 | 0.009 | 0.059 | 0.051 | 0.0012 | 7.04 | <0.001 |
| *≤*200m x 200m/*≥*300m x 300m | 0.007 | 0.004 | 0.054 | 0.051 | 0.0016 | 2.28 | 0.02 |
| *≤*300m x 300m/*≥*400m x 400m | 0.008 | 0.003 | 0.053 | 0.050 | 0.0023 | 1.42 | 0.16 |
| *Ethnic composition* |  |  |  |  |  |  |  |
| 0% | 0.009 | 0.007 | - | - | 0.0003 | 19.74 | <0.001 |
| (0-10%] | 0.005 | 0.002 | - | - | 0.0006 | 3.06 | <0.001 |
| >10% | 0.008 | 0.004 | - | - | 0.0006 | 6.47 | <0.001 |
| Covariates included | No | Yes | Yes | Yes | Yes | Yes | Yes |

**S9 Table.** **Statistical estimates per analytical setting on the subsample of natives who stay and move within Stockholm county**. *Note*: Estimates on the difference scale are computed both on the full sample (Raw) and on the matched sample (CEM-LPM). Predicted probabilities, standard errors, t-values and p-values are gauged using the matched sample.

*Agent-based model*

Our model is a first approximation to study the effects of social influence (SI) in residential out-mobility on Ethnic Residential Segregation (ERS). The decision has been to keep the model as simple as possible while still being informative about the research question of the main paper. The model is mainly based on Schelling's [10] spatial proximity model (SPM), in which households belonging to one of two groups, reds and greens, decide where they want to move according to the group share in their immediate spaces. If the share of same-group neighbors in households’ most proximate space is lower than 50%, households leave their current spot (a.k.a. the homophily rule). Moreover, households at the start of the simulation are randomly arranged in a grid, which causes ERS, our main macro-outcome of analysis, to be always low at the beginning of each simulation. Hence, our study aims at detailing how ERS deviates from this random state and changes as a result of households' interactions through time. We measure ERS using the Dissimilarity index [11].

To include SI in the model, we introduce an extra procedure to the SPM in which households that observe a neighbor leaving following the homophily rule figure out whether to follow the neighbor and move or to stay instead. The way we have implemented SI is so that we can vary its force, analogous to our concept of strength of SI in the paper. Thus, when the force of SI is zero, our model is basically the SPM, and households only move according to the homophily rule. Conversely, when the strength of SI is greater than zero, households' mobility is due to both the homophily rule and SI (i.e., SPM+SI model). Please, see the corresponding README file with instructions about how to open the model in NetLogo and how to reproduce our results.

**REFERENCES**

1. Dunning T. Improving causal inference: Strengths and limitations of natural experiments. Polit Res Q. 2008;61: 282–293. doi:10.1177/1065912907306470

2. Stuart EA. Matching methods for causal inference: A review and a look forward. Stat Sci. 2010;25: 1–21. doi:10.1214/09-STS313

3. Rosenbaum P, Rubin D. The central role of the propensity score in observational studies for causal effects. Biometrika. 1983;70: 41–55.

4. Ho D, Imai K, King G, Stuart EA. Matching as nonparametric preprocessing for reducing model dependence in parametric causal inference. Polit Anal. 2007;15: 199–236. doi:10.1093/pan/mpl013

5. Iacus SM, King G, Porro G. Multivariate matching methods that are monotonic imbalance bounding. J Am Stat Assoc. 2011;106: 345–361. doi:10.1198/jasa.2011.tm09599

6. Iacus S, King G, Porro G. cem: Software for Coarsened Exact Matching. J Stat Softw. 2009;30: 1–27.

7. Iacus SM, King G, Porro G. Causal inference without balance checking: Coarsened exact matching. Polit Anal. 2012;20: 1–24. doi:10.1093/pan/mpr013

8. Austin P. Samples, Balance diagnostics for comparing the distribution of baseline covariates between treatment groups in propensity-score matched. Stat Med. 2009;28: 3083–3107. doi:10.1002/sim

9. Pearl J. An introduction to causal inference. Int J Biostat. 2010;6: Article 7. doi:10.1177/0049124196024003004

10. Schelling TC. Dynamic models of segregation. J Math Sociol. 1971;1: 143–186. doi:10.1080/0022250X.1971.9989794

11. Taeuber K, Taeuber A. A practitioner’s perspective on the Index of Dissimilarity. Am Sociol Rev. 1976;41: 884–889.

1. Dunning T. Improving causal inference: Strengths and limitations of natural experiments. Polit Res Q. 2008;61: 282–293. doi:10.1177/1065912907306470

2. Stuart EA. Matching methods for causal inference: A review and a look forward. Stat Sci. 2010;25: 1–21. doi:10.1214/09-STS313

3. Rosenbaum P, Rubin D. The central role of the propensity score in observational studies for causal effects. Biometrika. 1983;70: 41–55.

4. Ho D, Imai K, King G, Stuart EA. Matching as nonparametric preprocessing for reducing model dependence in parametric causal inference. Polit Anal. 2007;15: 199–236. doi:10.1093/pan/mpl013

5. Iacus SM, King G, Porro G. Multivariate matching methods that are monotonic imbalance bounding. J Am Stat Assoc. 2011;106: 345–361. doi:10.1198/jasa.2011.tm09599

6. Iacus S, King G, Porro G. cem: Software for Coarsened Exact Matching. J Stat Softw. 2009;30: 1–27.

7. Iacus SM, King G, Porro G. Causal inference without balance checking: Coarsened exact matching. Polit Anal. 2012;20: 1–24. doi:10.1093/pan/mpr013

8. Austin P. Samples, Balance diagnostics for comparing the distribution of baseline covariates between treatment groups in propensity-score matched. Stat Med. 2009;28: 3083–3107. doi:10.1002/sim

9. Pearl J. An introduction to causal inference. Int J Biostat. 2010;6: Article 7. doi:10.1177/0049124196024003004

10. Schelling TC. Dynamic models of segregation. J Math Sociol. 1971;1: 143–186. doi:10.1080/0022250X.1971.9989794

11. Taeuber K, Taeuber A. A practitioner’s perspective on the Index of Dissimilarity. Am Sociol Rev. 1976;41: 884–889.
